# Responding to Young People’s Health Risks in Primary Care: A Cluster Randomised Trial of Training Clinicians in Screening and Motivational Interviewing

**DOI:** 10.1371/journal.pone.0137581

**Published:** 2015-09-30

**Authors:** Lena Sanci, Patty Chondros, Susan Sawyer, Jane Pirkis, Elizabeth Ozer, Kelsey Hegarty, Fan Yang, Brenda Grabsch, Alan Shiell, Helen Cahill, Anne-Emmanuelle Ambresin, Elizabeth Patterson, George Patton

**Affiliations:** 1 Department of General Practice, Melbourne Medical School, The University of Melbourne, 200 Berkeley St., Carlton, VIC, 3053, Australia; 2 Centre for Adolescent Health, Royal Children’s Hospital, 50 Flemington Rd., Parkville, VIC, 3052, Australia; 3 Department of Paediatrics, The University of Melbourne, VIC, 3010, Australia; 4 Murdoch Children’s Research Institute, 50 Flemington Rd., Parkville, VIC, 3052, Australia; 5 Melbourne School of Population and Global Health, Level 5, 207 Bouverie St., The University of Melbourne, VIC, 3010, Australia; 6 Division of Adolescent & Young Adult Medicine, University of California San Francisco, San Francisco, 94118, United States of America; 7 UCSF Office of Diversity and Outreach, University of California San Francisco, San Francisco, 94118, United States of America; 8 Centre of Excellence in Intervention and Prevention Science Limited, 15–31 Pelham St., P.O. Box 35, Carlton, VIC, 3053, Australia; 9 Youth Research Centre, Melbourne Graduate School of Education, The University of Melbourne, 100 Leicester St., Carlton, VIC, 3053, Australia; 10 Division Interdisciplinaire de santé des adolescents, Centre Hospitalier Universitaire Vaudois Lausanne Switzerland, Av. De Beaumont 48, CH-101, Lausanne, Switzerland; 11 Department of Nursing, Melbourne School of Health Sciences, The University of Melbourne, 161 Barry St., Carlton, VIC, 3053, Australia; National Center of Neurology and Psychiatry, JAPAN

## Abstract

**Objective:**

To evaluate the effectiveness of a complex intervention implementing best practice guidelines recommending clinicians screen and counsel young people across multiple psychosocial risk factors, on clinicians’ detection of health risks and patients’ risk taking behaviour, compared to a didactic seminar on young people’s health.

**Design:**

Pragmatic cluster randomised trial where volunteer general practices were stratified by postcode advantage or disadvantage score and billing type (private, free national health, community health centre), then randomised into either intervention or comparison arms using a computer generated random sequence. Three months post-intervention, patients were recruited from all practices post-consultation for a Computer Assisted Telephone Interview and followed up three and 12 months later. Researchers recruiting, consenting and interviewing patients and patients themselves were masked to allocation status; clinicians were not.

**Setting:**

General practices in metropolitan and rural Victoria, Australia

**Participants:**

General practices with at least one interested clinician (general practitioner or nurse) and their 14–24 year old patients.

**Intervention:**

This complex intervention was designed using evidence based practice in learning and change in clinician behaviour and general practice systems, and included best practice approaches to motivating change in adolescent risk taking behaviours. The intervention involved training clinicians (nine hours) in health risk screening, use of a screening tool and motivational interviewing; training all practice staff (receptionists and clinicians) in engaging youth; provision of feedback to clinicians of patients’ risk data; and two practice visits to support new screening and referral resources. Comparison clinicians received one didactic educational seminar (three hours) on engaging youth and health risk screening.

**Outcome Measures:**

Primary outcomes were patient report of (1) clinician detection of at least one of six health risk behaviours (tobacco, alcohol and illicit drug use, risks for sexually transmitted infection, STI, unplanned pregnancy, and road risks); and (2) change in one or more of the six health risk behaviours, at three months or at 12 months. Secondary outcomes were likelihood of future visits, trust in the clinician after exit interview, clinician detection of emotional distress and fear and abuse in relationships, and emotional distress at three and 12 months. Patient acceptability of the screening tool was also described for the intervention arm. Analyses were adjusted for practice location and billing type, patients’ sex, age, and recruitment method, and past health risks, where appropriate. An intention to treat analysis approach was used, which included multilevel multiple imputation for missing outcome data.

**Results:**

42 practices were randomly allocated to intervention or comparison arms. Two intervention practices withdrew post allocation, prior to training, leaving 19 intervention (53 clinicians, 377 patients) and 21 comparison (79 clinicians, 524 patients) practices. 69% of patients in both intervention (260) and comparison (360) arms completed the 12 month follow-up. Intervention clinicians discussed more health risks per patient (59.7%) than comparison clinicians (52.7%) and thus were more likely to detect a higher proportion of young people with at least one of the six health risk behaviours (38.4% vs 26.7%, risk difference [RD] 11.6%, Confidence Interval [CI] 2.93% to 20.3%; adjusted odds ratio [OR] 1.7, CI 1.1 to 2.5). Patients reported less illicit drug use (RD -6.0, CI -11 to -1.2; OR 0·52, CI 0·28 to 0·96), and less risk for STI (RD -5.4, CI -11 to 0.2; OR 0·66, CI 0·46 to 0·96) at three months in the intervention relative to the comparison arm, and for unplanned pregnancy at 12 months (RD -4.4; CI -8.7 to -0.1; OR 0·40, CI 0·20 to 0·80). No differences were detected between arms on other health risks. There were no differences on secondary outcomes, apart from a greater detection of abuse (OR 13.8, CI 1.71 to 111). There were no reports of harmful events and intervention arm youth had high acceptance of the screening tool.

**Conclusions:**

A complex intervention, compared to a simple educational seminar for practices, improved detection of health risk behaviours in young people. Impact on health outcomes was inconclusive. Technology enabling more efficient, systematic health-risk screening may allow providers to target counselling toward higher risk individuals. Further trials require more power to confirm health benefits.

**Trial Registration:**

ISRCTN.com ISRCTN16059206.

## Introduction

Rapid changes in global patterns of health have brought fresh attention to health risks arising in adolescence and young adulthood [1–3]. These are the peak years for the onset of mental disorders, injuries and reproductive health risks. There is also an emerging recognition that the seeds of later life non-communicable diseases are laid in health risks, such as substance use, obesity and low rates of physical exercise, established during these years [1, 2]. Tied to evidence that the health status of adolescents relative to younger children has improved little in high and middle income countries over the past five decades, there are growing calls for more effective responses [4].

Primary care has a potentially important role in responding to health risks in young people. Most adolescents and young adults in high and middle income countries attend primary care clinics at least annually [5] and the health risks facing adolescents frequently cluster [6], resulting in repeated opportunities to address multiple risks. Yet primary care has not hitherto figured prominently in prevention strategies targeting young people [2, 5]. One reason is that young people rarely present to clinicians primarily for assistance with their risky behaviours [7]. However youth report welcoming these discussions if raised sensitively by youth-friendly providers [7].

Although best practice guidelines continue to call for clinicians to use health care visits as an opportunity to screen for a range of health risks and intervene early or provide preventive health advice [8, 9], the evidence for this approach is scant [5]. There are many empirical challenges in primary care settings for research investigating the health outcomes of preventive screening. These challenges include achieving sample sizes large enough to detect meaningful effect sizes across multiple health outcomes; poor clinician adherence to intervention delivery; poor adolescent adherence to management plans; the long follow-up periods required to measure the adverse outcomes of risk taking, and; the difficulties of attributing differences in health outcomes to one clinical intervention when the causes of such health risks are complex and multifactorial [10–12]. Yet in spite of these challenges there are calls for more research to support policy and practice initiatives in this area [10, 11].

We identified only three earlier randomised trials of screening and counselling interventions addressing co-occurring health risk behaviours in primary care [13–15]. Two were targeted interventions testing counselling delivered in specialist primary care clinics for HIV affected youth [13] and pregnant youth [14]. Only one, a well care clinic, was conducted in general practice [15]. This study showed only modest improvements in intention to change risky behaviour. Other studies suggest that detection of risk is greater if screening occurs opportunistically during regular visits [16].

Our prior randomised controlled trial showed that training could change clinicians’ behaviour and improve relationships with simulated young patients in simulated general practice settings, but stopped short of examining whether this training could be implemented in real general practice settings and whether health outcomes could be improved as a result [17].

To address this gap in understanding the potential of primary care in youth health, we conducted a pragmatic cluster randomised trial [18] of a complex intervention delivered in routine general practice, that built on prior work [17, 19, 20]. The trial fulfils the criteria of pragmatic as defined by Zwarenstein and colleagues (2008) [18], namely: it is an effectiveness trial testing whether the intervention works when used in normal practice; participants are not highly selected, other than being in the appropriate age group; the intervention is applied flexibly as it would be in normal practice; the outcomes are directly relevant to clinicians, patients and funders; and the trial design has direct relevance to clinicians practicing in the usual setting in which the intervention would be implemented. Our intervention involved training sessions for clinicians and practice support staff in youth-friendly care, use of screening tools, feedback from patients, and consultation to practices around the introduction of health risk screening and counselling, opportunistically, with all young people who presented for any reason. The elements of the intervention were selected using evidence-based principles about effective clinician education and practice change [21]. Comparison clinicians received three hours of didactic teaching on youth-friendly care and health risk screening. We hypothesised that the intervention relative to the comparison arm would (1) increase clinician detection of health risk behaviours, emotional distress and abuse (through increased screening/discussion of risks with youth); (2) reduce at least one risk taking behaviour, amongst young people at three or 12 months post-consultation; (3) and would be acceptable to young people. The two time frames were included because individuals vary widely in their time to behaviour change [22] and also to capture whether any changes observed at three months were sustained at 12 months. In this paper we report on the main outcomes of the trial. Our intervention, with several interacting components, qualifies as a complex intervention [23] implemented in the real life complex system of primary care [24] and therefore, in keeping, has more than one primary outcome [23]. An economic evaluation was also conducted and parental and staff attitudes toward the intervention measured. These will be addressed in separate papers.

## Methods

### Study design and participants

The detail of our trial protocol has been previously published [25]. Briefly, we used a stratified cluster randomised design and enrolled general practices and their young patients between June 2007 and July 2011 (S1 File), consistent with CONSORT guidelines [18, 26, 27]. Data were collected on two distinct samples of young people from the same practices. First, a cross-sectional sample of young people presenting within a two week period to each participating practice was recruited prior to randomisation and these young people completed the ‘profile exit interview’ but were not followed up over time. The purpose of the ‘baseline profile sample’ was to obtain a snapshot of the demographic details of young people consulting with the clinician before practices were randomly allocated to their study arm status. The second sample formed the study cohort of young people recruited from the same practices post-randomisation, around three months post-intervention, and were followed up three and 12 months later to measure outcomes. The specific dates for the phases of the trial depicted in the S1 File are as follows: practice recruitment and recruitment of baseline profile sample and completion of the exit interview, 1^st^ June 2007 to 11^th^ February 2010; practice randomisation, 4^th^ July 2007 to 13^th^ April 2010; intervention training, 18^th^ July 2007 to 3^rd^ June 2010; cohort sample recruited into trial, 6^th^ September 2007 to 29^th^ July 2010; cohort sample completion of exit interview, 7^th^ September 2007 to 28^th^ August 2010; three month follow-up, 7^th^ January 2008 to 30^th^ November 2010; and 12 month follow-up, 9^th^ September 2008 to 8^th^ July 2011. The general practice was the unit of randomisation to minimise the risk of contamination if clinicians and practice staff were in contact with patients from both arms. Our primary outcomes were patient report of: (1) clinician detection during the consultation of at least one of six health risk behaviours (tobacco, alcohol, and illicit substance use, risks for sexually transmitted infection [STI] and unplanned pregnancy, and risks to road safety), and (2) change in one or more of the six health risk behaviours, at three months and at 12 months post-consultation. Secondary outcomes were young people’s likelihood of returning [28], trust in the clinician [29], and patient acceptance of a screening tool. We were also interested in whether training in a broad health risk screen that included mental health and safety would result in detection of more individuals with emotional distress and who had experienced fear or abuse in family or intimate partner relationships, and whether there would be any changes in emotional distress at three and 12 months post-consultation.

The intervention had two main components operating at the practice level: nine hours of experiential workshops and two practice visits (see S2 File for details of the intervention). Comparison clinicians received one three-hour seminar on youth-friendly care including recommendations to discuss health risks with young people.

All practices in 15 urban (N = 1213 practices) and eight regional (N = 278 practices) divisions in Victoria, Australia [30], were exposed to advertisements for expressions of interest in being involved in the research either via newsletters, direct mail out or phone calls. Eligible practices required at least one clinician (general practitioner [GP] and/or practice nurse [PN]) to take part. Young people were eligible if aged between 14–24 years and attending a study clinician, and excluded if severely physically or mentally unwell, unable to read or speak English, or if judged an immature minor without parental consent [25].

Clinicians for both the baseline profile sample and cohort sample (S1 File) were instructed to assess all their patients aged 14–24 years for eligibility and to ask eligible patients for permission to forward their telephone number to researchers to hear more about the study. Clinicians also handed patients an information pack including a health issues service directory and completed an encounter form on each patient which included presenting reason, diagnoses and management. Intervention clinicians documented use of the study designed screening tool or another form of risk assessment. Research assistants (RAs) phoned patients as close as possible to their consultation to obtain informed consent and proceed with a Computer Assisted Telephone Interview (CATI); the ‘exit interview.’ Research staff monitored the number of 14–24 year old patients attending the practice weekly compared to the number assessed by GPs for eligibility and found that clinicians in both arms failed to consecutively approach every eligible patient, thereby considerably lengthening the time required to recruit a sufficient number of young people needed for the cohort sample to be followed up at three and 12 months. Hence from 28/7/2009, RAs were placed in the remaining eight intervention and seven comparison practices to systematically approach eligible young people, following the same procedure as the clinicians [25]. We documented the changes in patients approached per week before and after the introduction of RAs for the first six of the practices to receive RAs (three intervention and three comparison). It had taken a total of 798 days for clinicians to approach 61 patients, (0.5 patients per week) compared to 134 days in these same practices for RAs to approach 126 patients (6.6 patients per week).

Outcome measures are summarised in the supporting information (S3 File) and detailed elsewhere [25]. The six health risk behaviours, emotional distress and abuse were defined using a combination of expert clinical opinion, instrument cut-off scores and national longitudinal population data (S4 File) [31, 32–34]. Clinicians poorly documented their assessments of the young person’s risk status in encounter forms. Instead, we defined a clinician detected risk if the young person reported in the exit interview that they had discussed the risk with their clinician and they also reported that they were engaging in that risk.

### Randomisation and masking

An independent statistician, otherwise not involved with the study, generated a random allocation sequence in STATA [35] stratified by postcode level advantage-disadvantage socio-economic scores [36] (dichotomised into low versus middle/high tertiles) and type of practice (private billing, free national health funded, and community health centres) forming six strata. Block randomisation with fixed block sizes of two was used within strata [25]. Practices were assigned unique identifier codes so that the study statistician (PC) analysing the trial data was masked to study group allocation. The independent statistician notified the trial coordinator (BG) of the practice’s study arm allocation, who then notified the practices about their allocation after the baseline profile data were collected on the sample of young people attending the practice.

The allocation sequence was fully protected until the 12-month follow-up was completed.

The different training received in intervention and comparison arms precluded masking practices to their study allocation; to obtain fully informed consent for participation, clinicians required information about the potential commitment to training in both study arms. The RAs recruiting and consenting patients and conducting CATIs were masked to practice allocation. Patients were not informed of the practice allocation in any researcher communication.

### Statistical analysis

Sample size was calculated for clinician detection of one or more risky health behaviours assuming 80% power and 5% significance level for a two-sided test. Due to slow clinician recruitment, our revised hypothesised effect size (detailed elsewhere [25]), was 12·5% for clinician detection of a risk behaviour. We assumed that 40% of youth attending general practice have psychosocial risks and that trained GPs at best will detect 60% of these with interview alone [37], equivalent to 24% of all presenting youth. We expected intervention clinicians to detect 91% (i.e. a further 31% to the 60% in the comparison arm) of risk taking youth, equivalent to about 36.5% of all presenting youth. To detect this 12·5% difference in detection between the study arms we required 20 practices and 360 youth (18 per practice) in each arm, assuming an intra-clinic correlation (ICC) of 0·04 [38]. This sample size was also sufficient to detect a 15% difference between the two study arms on the alcohol use behaviour assuming 41% of youth use alcohol in the comparison arm and an ICC of 0.07 [25, 39] with 80% power (5% significance level, 2 sided test). Smaller risk differences for individual health risks between arms could be detected for less prevalent health risk behaviours (e.g. substance abuse = 38%, tobacco use = 24%) [39] and for less conservative values of the ICC for health risks (0.01 and 0.04). See Table 2 in protocol paper for details [25]. The total sample size was increased to 1,260 youth (30 per practice) from 42 practices to allow for loss of practices [40] and for 40% loss to follow-up of young people over 12 months [41].

Descriptive statistics were used to compare practice, clinician and young people’s characteristics between study arms. ICCs for outcomes were calculated using one-way analysis of variance. Analyses used an intention-to-treat approach. First, a complete case analysis that included all available data was conducted which was valid under the assumption that the data are missing completely at random. Sensitivity analyses were then conducted to assess the robustness of the missing data assumption using multilevel multiple imputation approach (See S5 File for full details). Linear regression was used to estimate the difference in mean outcome between arms for continuous outcomes. Generalised linear model, specifying the binomial family with either the identity, log or logit link function, was used to estimate the risk difference (RD), risk ratio (RR) and odds ratio (OR) for binary outcomes, respectively. Relative risk reduction (RRR) was calculated using (1-RR) x100. Multivariable regression was used to adjust for the stratification variables, patient recruitment by the clinician or RA, patient sex and age and baseline risk factor status where appropriate. Estimates were reported with 95% confidence intervals (CI) and p-values. Generalised estimating equations with robust standard errors were used with all regression analyses to adjust for the correlation of outcomes within practices and where applicable repeated outcome measures over time, provided the estimated ICC for the fitted model was non-negative. Analyses were conducted using Stata 13 [35].

### Ethics approval

The study obtained ethics approval from the Health Sciences Subcommittee of the University of Melbourne Human Research Ethics Committee for the project ‘Health risk screening and counselling of young people in primary care: a cluster randomised controlled trial’, Ethics ID 0709280, with amendments 0709280.1, 0709280.2, 0709280.3 and 0709280.4. The Melbourne Human Research Ethics Committee approved all our consenting processes which were as follows (any further detail or the applications themselves will be made available on request). Clinicians or RAs approaching patients for permission to pass their preferred telephone number to CATI RAs for the full consenting procedure noted on recruitment ledgers that patients consented to provide their preferred telephone number and a consent form was also signed for this. This was classified as minimal risk research therefore we were able to consider the consent of mature minors, without guardian consent. When the patient was a minor (14 to 18 years) and was attending without a guardian and did not want their guardian to be aware of their visit, consent for this process was accepted only if the clinician assessed the patient to be a mature minor. If the minor was attending with a guardian, written and verbal guardian consent was also obtained. If the minor was not judged to be mature and was either alone or refusing to inform guardians, he/she was excluded from the study. A formal consenting procedure was undertaken by the CATI RAs who outlined the details of participation on the phone because this process was felt to take too long to explain and be too disruptive to flow of patients to perform in the practice and also patients would be free to consider participation or not without any potential influence from practice staff. This consent was therefore verbal consent and was recorded formally in the CATI process on the web-based survey instrument. Participants aged 14–18 years, who were deemed to be mature minors by the clinician, were also consented in this way. CATI RAs also obtained and recorded verbal consent from guardians of 14–18 year olds who had attended the practice with the adolescent. Verbal consent was again checked and recorded in the survey from each previously consented patient by the CATI RA before proceeding with the actual interview itself and before each of the three and 12 month follow-up interviews, which were also performed by telephone (usually mobile phone).

## Results

### Trial flow and participant characteristics

**Fig 1:**
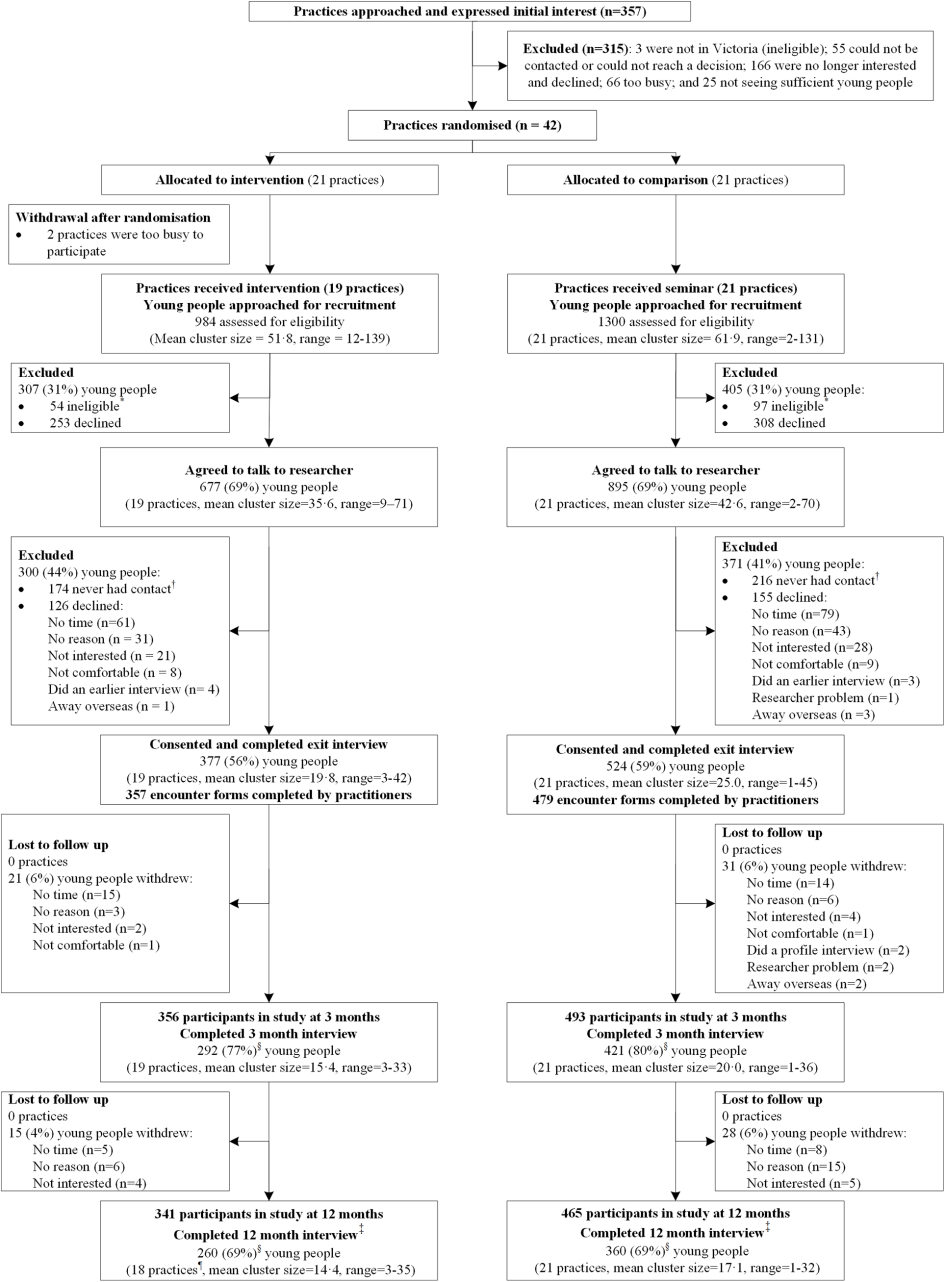
Trial Profile of practices and cohort sample. * Young people were ineligible if they were: unable to speak English; physically or mentally unwell; or, under 18 years old, judged by clinician to be incompetent to make an informed decision for participation in minimal risk research and unable or unwilling to obtain parental consent. † Never had contact (wrong phone number, too many attempted phone calls, or responded more than three weeks post consultation and were therefore too delayed to participate). ‡ A small number of young people completed the baseline survey and 12 month follow-up CATI but did not complete the three month CATI because they were too busy, away or not contactable during this time. § Denominator used to calculate the percentage is the total number of young people who consented and completed exit interview. ¶ One practice had no young people complete the 12-month CATI.

We approached 357 practices that expressed interest in the trial until 42 consented to participate [25]. Fig 1 shows the trial profile for the young people recruited post randomisation and followed up at three and 12 months. Two intervention arm practices withdrew post-randomisation but pre-intervention, leaving 19 intervention (53 clinicians, 377 patients) and 21 comparison (79 clinicians, 524 patients) practices. No further practices withdrew but one intervention practice had no young people complete the 12 month CATI.

Attrition rates of young people over time were similar between the two arms (Fig 1). The demographic measures and self-reported health risks of young people lost to follow-up did not differ between arms (S5 File). Baseline characteristics of clinicians were similar in both arms (Table 1) except intervention practices tended to be smaller with older and fewer female GPs. Around 60% of GPs in both arms had prior training in young people’s health, but more intervention PNs reported prior training. Compared to Australian general practices, the proportion of urban practices was greater in our sample (80% vs 72%) [42], as was the proportion of GPs that were female (51% vs 39%) [43] and under 45 years old (49% vs 28%) [44]. There were fewer solo practices in our sample (15% vs 21%) [45].

**Table 1:** Baseline characteristics of general practices, general practitioners (GPs) and practice nurses (PNs).

	Intervention		Comparison	
	n	(%)	n	(%)
**Practice**	**19**		**21**	
**Practice billing**				
Bulk-billing	5	(26)	7	(33)
Private-billing	11	(58)	13	(62)
Community Health Centre	3	(16)	1	(5)
**SEIFA Advantage** *	15	(79)	17	(81)
**Urban location** ^†^	17	(89)	15	(71)
**Participating GPs per practice**				
0^‡^	1	(5)	0	(0)
1	10	(53)	8	(38)
2	6	(32)	4	(19)
3	0	(0)	3	(14)
4–8	2	(11)	6	(29)
**Participating PNs per practice**				
0	7	(37)	8	(38)
1	7	(37)	8	(38)
2	0	(0)	4	(19)
3–4	5	(26)	1	(5)
**General Practitioners** ^§^	**31**	** **	**59**	** **
**Age group (years)**				
25–34	1	(3)	7	(13)
35–44	9	(30)	26	(47)
45–54	15	(50)	15	(27)
55–64	5	(17)	5	(9)
65 or over	0	(0)	2	(4)
**Female**	14	(45)	32	(54)
**Graduated in Australia**	22	(73)	41	(76)
**Year of graduation**				
1960–1969	0	(0)	2	(4)
1970–1979	7	(23)	6	(11)
1980–1989	15	(50)	19	(35)
1990–1999	6	(20)	21	(38)
2000–2009	2	(7)	7	(13)
**Prior adolescent health training**	18	(62)	34	(62)
**Practice Nurses** ^||^	**22**		**20**	
**Age group (years)**				
25–34	5	(26)	2	(13)
35–44	8	(42)	6	(40)
45–54	5	(26)	7	(47)
55–64	1	(5)	0	(0)
**Graduated in Australia**	17	(89)	14	(93)
**Year of graduation**				
1960–1969	1	(5)	0	(0)
1970–1979	3	(16)	4	(27)
1980–1989	5	(26)	6	(40)
1990–1999	6	(32)	3	(20)
2000–2009	4	(21)	2	(13)
**Prior adolescent health training**	10	(50)	3	(20)

Totals vary due to missing responses.

* Australian Bureau of Statistics [36]

† Commonwealth Department of Health and Aged Care [46]

^‡^ One community health centre consisted of two PNs only and would refer to the GP when needed

^§^ 3 GPs in the intervention arm and 5 in comparison arm had some missing demographic data

^||^ All 42 PNs were female; 3 PNs in the intervention arm and 5 in comparison arm had some missing demographic data.

Participation in their respective training was generally high for clinicians in both arms. Of the 31 intervention GPs, 29 (94%) attended at least one of the three training workshops and 74% attended all three, whereas 47 of 59 (80%) comparison GPs attended their single training session. Of the 22 intervention PNs, 16 (73%) attended any training and 11 (50%) attended all three sessions. Fifteen of 20 (75%) comparison PNs attended their single three hour seminar. Sixteen of 19 (84%) intervention practices took up training for practice support staff (PSS). All 19 intervention practices received two practice visits, including feedback of patient data.

The distribution of young people’s characteristics and health risks (Table 2) was similar between the profile (pre-randomisation, N = 389) and cohort exit interview (post-randomisation, N = 901) samples, and between the study arms for each sample. The exceptions where the intervention arm differed from the comparison were a higher proportion of patients in both samples aged 18–24 years, fewer in the cohort sample born in Australia, and more recruited by the RA. Overall about 87% of participants in both study arms reported having at least one of the six health risk behaviours at the exit interview, the most prevalent being road risks and then tobacco and alcohol use in the last 12 months. Similar to Australian national data [47] the most common reasons for presentation were for physical health issues, a checkup or administrative processes.

**Table 2:** Young people's characteristics in the baseline profile (N = 389) and cohort (N = 901) samples by study arm.

	Profile				Cohort*					
	Intervention		Comparison		Intervention			Comparison		
	**n**	**(%)**	**n**	(%)	**n**	**(%)**		**n**	**(%)**	
**Total number of young people**	169		220		377			524		
**Females**	120	(71.0)	168	(76.4)	287	(76.1)		395	(75.4)	
**Age group**										
14 to 15 years	18	(10.7)	22	(10.0)	39	(10.3)		62	(11.8)	
16 to 17 years	20	(11.8)	40	(18.2)	37	(9.8)		85	(16.2)	
18 to 24 years	131	(77.5)	158	(71.8)	301	(79.8)		377	(71.9)	
**Born in Australia**	143	(85.1)	196	(89.1)	284	(75.3)		472	(90.4)	
**Recruited by GP (versus RA)**	—		—		249	(66.0)		417	(79.6)	
**Employment and Study Status**										
Studying only	42	(25.0)	57	(26.2)	104	(27.7)		133	(25.5)	
Working only	32	(19.1)	60	(27.5)	82	(21.8)		141	(27.0)	
Both working and studying	84	(50.0)	79	(36.2)	163	(43.4)		210	(40.2)	
Neither working or studying	10	(6.0)	22	(10.1)	27	(7.2)		38	(7.3)	
**Attended usual practice?**										
Yes	126	(74.6)	190	(86.4)	269	(71.7)		421	(80.7)	
No	34	(20.1)	20	(9.1)	76	(20.3)		82	(15.7)	
Don't have one	9	(5.3)	10	(4.6)	30	(8.0)		19	(3.6)	
**Saw clinician on own**	130	(79.3)	157	(71.7)	309	(82.8)		375	(71.7)	
**At last consultation did you see. . .**										
General practitioner	132	(78.6)	192	(87.3)	296	(78.5)		466	(88.9)	
Practice nurse	8	(4.8)	16	(7.3)	34	(9.0)		19	(3.6)	
Both	28	(16.7)	12	(5.5)	47	(12.5)		39	(7.4)	
**Number of visits with GP in last 12 months**										
First visit	44	(27.5)	62	(30.4)	95	(27.8)		140	(27.8)	
1–2 times	42	(26.3)	50	(24.5)	93	(27.2)		128	(25.4)	
3–4 times	29	(18.1)	38	(18.6)	68	(19.9)		89	(17.7)	
5–6 times	18	(11.3)	12	(5.9)	38	(11.1)		60	(11.9)	
7 or more times	27	(16.9)	42	(20.6)	48	(14.0)		86	(17.1)	
**Number of visits with PN in last 12 months**										
First visit	19	(54.3)	14	(50.0)	51	(63.7)		36	(63.2)	
1–2 times	4	(11.4)	9	(32.1)	16	(20.0)		13	(22.8)	
3–4 times	3	(8.6)	4	(14.3)	8	(10.0)		4	(7.0)	
5–6 times	1	(2.9)	1	(3.6)	2	(2.5)		3	(5.3)	
7 or more times	8	(22.9)	0	(0)	3	(3.8)		1	(1.8)	
**Health risks**	**n**	**(%)**	**n**	(%)	**n**	**(%)**	**ICC** ^†^	**n**	**(%)**	**ICC** ^†^
**At least one health risk (of 6)**	153	(90.5)	199	(90.7)	329	(87.3)		456	(87.2)	
Tobacco smoking (last 12 months)	76	(45.2)	91	(41.7)	140	(37.2)	0.021	210	(40.4)	0.017
Alcohol use (last 12 months)	74	(47.7)	86	(41.4)	154	(42.4)	0.048	210	(41.8)	0.020
Illicit drug use (last 12 months)	42	(24.9)	57	(26.0)	95	(25.2)	0.071	144	(27.5)	0.017
Risk of sexually transmitted infection	35	(21.1)	58	(27.1)	70	(18.7)	0.039	92	(17.7)	0.011
Risk of unplanned pregnancy	30	(18.0)	33	(15.6)	53	(14.2)	—	61	(11.9)	0.019
One or more road safety risks	136	(80.5)	180	(81.8)	301	(79.8)	—	408	(78.0)	0.0003
**Emotional distress (last month)**	63	(37.3)	78	(35.5)	121	(32.1)	0.032	143	(27.4)	0.063
**Fear or abuse in relationships (last 12 months)** ^‡‡^	40	(29.0)	32	(19.4)	69	(21.8)	—	92	(22.4)	0.059
**Number of encounters recorded by clinician**	155	(91.7)	196	(89.1)	348	(92.3)		474	(90.5)	
**Reason for presentation** ^‡^										
Physiological, checkup or administration	127	(81.9)	154	(78.6)	277	(79.6)		394	(83.1)	
Sexual or reproductive health	31	(20.0)	43	(21.9)	77	(22.1)		91	(19.2)	
Psychosocial issues	18	(11.6)	18	(9.2)	28	(8.0)		40	(8.4)	

Abbreviations: RA = Research Assistant; GP = General Practitioner; PN = Practice Nurse; Totals vary due to missing responses; Note: Profile refers to the cross-sectional sample of young people recruited from practices prior to randomisation whereas the cohort sample were the subjects of the trial recruited post randomisation and followed up at three and 12 months. see S1 File.

* 312 young people in the intervention arm and 485 young people in comparison arm were seen by the study enrolled GP only; 65 young people in the intervention arm and 34 young people in comparison arm were seen by the study enrolled PN only; 5 young people in the comparison arm were seen by both the study enrolled GP and PN.

† Intra-cluster correlation (ICC) estimated for health risk factors of cohort at exit interview for each arm using one way analysis of variance; ICC values not shown were truncated at zero.

^‡‡^ Sensitive question only asked of young people aged 17 years old or greater (Cohort: N = 316 in intervention group and N = 411 in comparison group; Profile: N = 138 in intervention group and N = 165 in comparison group).

‡ Young people could present with multiple reasons, 90% young people present with one reason and 10% with two or three reasons for encounter coded by ICPC-2 [48].

### Clinicians’ detection of health risks in young people

Overall, intervention clinicians had more discussions with young people about their health risks (60%, 222/372 vs 53%, 272/516) and were more likely to discuss a greater number of health risks with each young person than comparison clinicians (S1 Table). In particular, they were more likely to discuss tobacco, alcohol and illicit drug use, road risks, and fear and abuse in relationships (S2 Table). There was no evidence to support differences between arms in discussion of sexual health risks and emotional distress.

Table 3 shows the proportion of risk factors that may have been detected by the clinicians when they discussed a risk factor and the young person also reported engaging in the risk. Intervention clinicians may have detected a higher proportion of young people with at least one of the six health risks than the comparison clinicians (unadjusted risk difference [RD] 11.6%, 95%CI: 2.93% to 20.3%) and is in line with our hypothesised risk difference of 12.5%. This difference between study arms seems to be driven mainly by increased detection of alcohol use and road risks. Although nearly 80% of young people reported having at least one of the road risks in both arms (Table 2), only 10.2% (38/372) of young people reported discussing road risks with the intervention clinicians and fewer in the comparison group (1.4%, 7/515) (S2 Table) and this is reflected in the small proportion of young people detected with the road risks; 9.4% in the intervention and 0.6% in the comparison arms.

**Table 3:** Clinicians detection of risk-taking behaviours of the young person at the consultation measured at the “Exit interview” in the cohort sample of young people (N = 901).

	Intervention (N = 377)			Comparison(N = 524)			Unadjusted			Adjusted^§^		
Detection of health risks*	n	(%)	ICC^†^	n	(%)	ICC^†^	OR^‡^	(95% CI)	*P*	OR^‡^	(95% CI)	*P*
**At least one health risk**	**143**	**(38.4)**	**0.035**	**138**	**(26.7)**	**0.042**	**1.69**	**(1.14 to 2.52)**	**0.01**	**1.65**	**(1.11 to 2.46)**	**0.01**
Tobacco use	60	(16.2)	0.028	74	(14.5)	0.018	1.18	(0.75 to 1.84)	0.48	1.12	(0.74 to 1.72)	0.59
Alcohol use	54	(15.1)	0.061	38	(7.7)	—	2.12	(1.26 to 3.57)	0.005	2.29	(1.25 to 4.20)	0.01
Illicit drug use	19	(5.1)	0.027	20	(3.9)	0.015	1.34	(0.67 to 2.68)	0.41	1.27	(0.64 to 2.49) **	0.50
Sexual health												
*Contraception*	27	(7.4)	0.002	33	(6.5)	0.023	1.13	(0.62 to 2.05)	0.69	0.97	(0.55 to 1.69) **	0.90
*Protection from STIs*	27	(7.3)	—	36	(7.0)	0.025	1.04	(0.59 to 1.82)	0.90	1.02	(0.57 to 1.81)	0.95
Road and driving risks	35	(9.4)	0.078	3	(0.6)	—	19.5	(4.41 to 86.2)	0.0001	19.6	(4.35 to 88.5)	0.0001
Emotional distress	58	(15.7)	0.109	62	(12.0)	0.016	1.37	(0.77 to 2.4)	0.29	1.37	(0.82 to 2.37)	0.23
Fear or abuse in relationships^††^	9	(2.9)	—	1	(0.2)	—	12.0	(1.51 to 95)	0.02	13.8	(1.71 to 111)**	0.01

Abbreviations: OR = Odd ratio; CI = Confidence Interval; *P* = P-value; STIs = sexually transmitted infections; Totals vary due to missing responses.

* Detection of risk factors: YP reported that the issue was raised and/or discussed during the last consultation and reported having the health risk; issues discussed at previous consultations were coded as “No.”

§ Estimated OR adjusted for SES of the practice location, practice billing type, sex and age of young people, recruitment method of young people.

† Intra-cluster correlation (ICC) estimated using one way analysis of variance; ICC values not shown were truncated at zero.

‡ Estimated OR calculated using marginal logistic regression using generalised estimating equations with robust standard errors to adjust for clustering at the clinic level.

** Analysis does not adjust for clustering at the clinic level because estimated ICC for fitted model was negative.

^††^ Sensitive question only asked of young people aged 17 years old or greater (N = 313 in intervention group and N = 405 in comparison group).

### Young people’s health outcomes

Young people’s risk taking behaviour and emotional distress at three and 12 month follow-up post-consultation are reported in Table 4. A smaller proportion of youth in the intervention arm versus comparison arm reported illicit drug use in the last month at the three month (9% vs. 15%) and 12 month (10% vs. 16%) follow-ups. This corresponded to an unadjusted RRR in intervention compared to the comparison arm of 39% (95% CI 7% to 60%) and 40% (95% CI 7% to 62%) at three and 12 months respectively. There were also fewer intervention youth reporting risks for STI at three months (14% vs. 20%) and for unplanned pregnancy at 12 months (6% vs. 10%), corresponding to unadjusted RRRs in the intervention compared to comparison arm of 28% (95% CI -2% to 49%) and 45% (95% CI 0.5% to 69%) respectively. Analyses using multiple imputation for missing data for young people’s health risks gave similar results, see Table 5.

**Table 4:** Young people's health risks, three and 12 months post-intervention in the cohort sample of young people.

	Intervention			Comparison					Unadjusted			Adjusted^‡^		
	N	n	(%)	N	n	(%)	RD*	(95% CI)	OR^†^	(95% CI)	*P*	OR^†^	(95% CI)	*P*
**Tobacco smoking (last month)** ^§^														
3 months	292	68	(23·3)	420	111	(26·4)	-3·0	(-10·1 to 4·1)	0·91	(0·62 to 1·33)	0·63	0·95	(0·58 to 1·54) **	0·82
12 months	260	56	(21·5)	359	93	(25·9)	-3·2	(-12·3 to 5·9)	0·85	(0·52 to 1·38)	0·50	0·81	(0·49 to 1·32) **	0·40
**Alcohol use (last month)** ^§^														
3 months	286	96	(33·6)	420	168	(40·0)	-6·3	(-15·2 to 2·5)	0·76	(0·51 to 1·13)	0·18	0·69	(0·46 to 1·02) **	0·07
12 months	254	86	(33·9)	355	127	(35·8)	-1·8	(-11·3 to 7·7)	0·93	(0·60 to 1·43)	0·73	0·94	(0·62 to 1·41) **	0·76
**Illicit drug use (last month)** ^§^														
3 months	291	27	(9·3)	420	64	(15·2)	-6·0	(-10·7 to -1·2) **	0·57	(0·36 to 0·90)	0·02	0·52	(0·28 to 0·96) **	0·04
12 months	260	25	(9·6)	360	58	(16·1)	-6·5	(-11·7 to -1·3) **	0·55	(0·33 to 0·92)	0·02	0·63	(0·35 to 1·12) **	0·11
**Risk of STI (last 3 months)**														
3 months	289	41	(14·2)	418	82	(19·6)	-5·4	(-11·0 to 0·1) **	0·68	(0·47 to 1·00)	0·05	0·66	(0·46 to 0·96)	0·03
12 months	257	28	(10·9)	357	47	(13·2)	-2·2	(-8·5 to 4·0)	0·80	(0·43 to 1·49)	0·48	0·76	(0·43 to 1·34)	0·34
**Risk of unplanned pregnancy (last 3 months)**														
3 months	290	22	(7·6)	413	33	(8·0)	-0·04	(-4·4 to 3·6) **	0·94	(0·56 to 1·59)	0·82	0·91	(0·52 to 1·61) **	0·75
12 months	256	14	(5·5)	358	35	(9·8)	-4·4	(-8·7 to -0·1)	0·53	(0·28 to 1·00)	0·05	0·40	(0·20 to 0·80) **	0·01
**One or more road safety risks**														
3 months	292	211	(72·3)	420	297	(70·7)	1·5	(-5·2 to 8·3) **	1·03	(0·75 to 1·42)	0·85	1·09	(0·78 to 1·54)	0·61
12 months	260	187	(71·9)	360	268	(74·4)	-2·9	(-10·5 to 4·8)	0·84	(0·58 to 1·23)	0·37	0·79	(0·53 to 1·18)	0·25
**Emotional Distress (last month)**														
3 months	292	54	(18·5)	421	73	(17·3)	1·2	(-5·0 to 7·5)	1·09	(0·69 to 1·70)	0·72	1·07	(0·71 to 1·63)	0·74
12 months	260	50	(19·5)	360	55	(15·3)	3·6	(-4·3 to 11·5)	1·30	(0·75 to 2·24)	0·35	1·24	(0·71 to 2·15)	0·45

Abbreviations: RD = Risk difference; OR = Odd ratio; CI = Confidence Interval; *P* = P-value; STI = sexually transmitted infection; Discrepancies in totals due to missing responses.

‡ Estimated OR adjusted for socio-economic status of the practice location, practice billing type, sex and age of young people, recruitment method of young people and where indicated long term health risks measured at exit interview.

* Estimated RD calculated using generalised linear regression with identity link function using generalised estimating equations with robust standard errors to adjust for clustering.

† Estimated OR calculated using marginal logistic regression using generalised estimating equations with robust standard errors to adjust for clustering at the clinic level.

§ Adjusted for presence of this health risk in the 12 months preceding recruitment (long term health risk), measured by self-report at exit interview; Note: Illicit drug use includes cannabis use or/and other illicit drugs.

** Not adjusted for clustering at the clinic level because the estimated ICC for fitted model was negative.

**Table 5:** Young people's health risks, three and 12 months post-intervention in the cohort sample of young people with multilevel multiple imputation for missing data (N = 901).

	Intervention (N = 377)	Comparison (N = 524)	Unadjusted			Adjusted^†^		
	%	%	OR*	(95% CI)	*P*	OR*	(95% CI)	*P*
**Tobacco smoking (last month)** ^‡^								
3 months	23.5	25.8	0.91	(0.66 to 1.24)	0.54	0.90	(0.62 to 1.31)	0.60
12 months	21.8	27.1	0.78	(0.54 to 1.12)	0.18	0.78	(0.55 to 1.12)	0.18
**Alcohol use (last month)** ^‡^								
3 months	34.1	39.0	0.81	(0.59 to 1.12)	0.21	0.77	(0.55 to 1.06)	0.11
12 months	32.1	34.7	0.90	(0.64 to 1.25)	0.52	0.84	(0.61 to 1.15)	0.28
**Illicit drug use (last month)** ^‡^								
3 months	9.3	15.3	0.57	(0.37 to 0.88)	0.01	0.55	(0.33 to 0.90)	0.02
12 months	10.1	15.7	0.60	(0.39 to 0.92)	0.02	0.61	(0.38 to 0.97)	0.04
**Risk of STI (last 3 months)**								
3 months	14.3	19.2	0.71	(0.52 to 0.95)	0.02	0.70	(0.48 to 1.03)	0.07
12 months	10.3	12.6	0.80	(0.48 to 1.35)	0.41	0.79	(0.51 to 1.24)	0.31
**Risk of unplanned pregnancy (last 3 months)**								
3 months	7.0	8.2	0.84	(0.52 to 1.37)	0.49	0.85	(0.50 to 1.42)	0.53
12 months	6.9	10.2	0.65	(0.40 to 1.04)	0.07	0.53	(0.30 to 0.94)	0.03
**One or more road safety risks**								
3 months	72.6	71.0	1.07	(0.80 to 1.41)	0.66	1.08	(0.79 to 1.47)	0.65
12 months	71.4	73.9	0.87	(0.64 to 1.19)	0.38	0.81	(0.59 to 1.11)	0.19
**Emotional Distress (last month)**								
3 months	17.9	17.3	1.04	(0.71 to 1.53)	0.83	1.00	(0.70 to 1.41)	0.99
12 months	18.1	17.1	1.07	(0.72 to 1.59)	0.75	1.04	(0.70 to 1.57)	0.83

Abbreviations: OR = Odd ratio; CI = Confidence Interval; *P* = P-value; STI = sexually transmitted infection

†Estimated OR adjusted for socio-economic status of the practice location, practice billing type, sex and age of young people, recruitment method of young people and where indicated long term health risks measured at exit interview

* Estimated OR calculated using marginal logistic regression using generalised estimating equations with robust standard errors to adjust for clustering at clinic level

‡ Adjusted for presence of this health risk in the 12 months preceding recruitment (long term health risk), measured by self-report at exit interview; Note: Illicit drug use includes cannabis use or/and other illicit drugs.

### Secondary outcomes

The likelihood of returning for future visits and rating of trust in their clinician were the same in both arms; with both measures rating highly (S6 File). There was no evidence to support differences between arms in discussion (S2 Table) or detection of emotional distress (Table 3) or in presence of emotional distress at three or 12 months post-consultation (Tables 4 and 5). About 22% of young people 17 years and older reported fear or abuse in a relationship in both arms at the exit interview (Table 2), but this was seldom discussed during the consultation (7.2% (23/318) in intervention and 1% (4/413) in comparison arms (S2 Table).

The method of psychosocial health risk screening adopted by intervention clinicians was recorded in encounter forms for 75% (263/352) of recruitment consultations. The ‘pragmatic’ nature of the intervention was evident in that the study-designed screening tool was used in 30% (106/352) of consultations in the eight clinics that adopted the tool, while in 43% (151/352) of consultations, clinicians reported they screened verbally using the HEADSS framework [49]. Of the young people who responded that they completed a ‘lifestyle questionnaire’ in the intervention arm, 89% (93/105) rated it a ‘good idea’, 11% (12/105) were ‘unsure’ and no-one rated it a ‘bad idea’.

## Discussion

In this study we set out to test whether implementation of a complex intervention to encourage screening/discussion of psychosocial issues with all young people presenting and responding to health risk behaviours with motivational interviewing approaches in primary care might favourably shift adolescent health risks, including those for later life non-communicable disease. The intervention, with evidence-based components in clinician and youth behaviour change, compared to the didactic training delivered in the comparison arm, changed the interaction between clinicians and their young patients such that it included greater discussion of health risk behaviours and abuse in relationships, which contribute to disease burden, and may have resulted in greater detection of these health risks. However, there were still around 40% of consultations where none of the six common health risk behaviours were discussed. There was some evidence for a reduction in illicit drug use and taking precautions against STIs at three months and against unplanned pregnancy at 12 months in the intervention arm compared to comparison arm. Differences between arms were not however found across other health risks particularly emotional distress and road risks. There was low probability of harm from the intervention, given that youth in both arms highly rated their likelihood of returning and trust in their clinician, and that, consistent with other research [50], the majority supported the screening tool.

In spite of the empirical challenges in measuring health outcomes from preventive interventions in primary care, our study has strengths. It is the first pragmatic cluster randomised trial of a clinician training intervention targeting prevention for multiple health risks in young people in the complex setting of primary care where they are most likely to present for health care in many countries. Prior studies of training and tools promoting screening and counselling during planned well care visits in general paediatric clinics showed increased paediatrician discussion of health risks; however these were not randomised controlled trials [20, 51]. Two of three trials of behavioural interventions addressing multiple health risks in primary care settings have occurred in specialist primary care (antenatal [14] and HIV [13]), and have only enrolled participants with certain risk factors, hence the ‘screening component’ was conducted by the researchers and the interventions more targeted [13, 14]. These trials reported that more intervention participants had resolving health risks over time [14] or remained in a low risk group over time [13]. The only other trial in general practice invited 16 year olds to attend the practice for well care visits to discuss health risks with PNs [15]. Our trial is the first to test the combination of clinician screening and counselling delivered opportunistically to young people attending community-based general practice, where clinicians consult across the life course, and during consultations where young people present mainly for acute physical reasons rather than well care visits.

Further strengths of our study include randomising practices instead of young people to minimise the risk of contamination where the clinician may inadvertently expose the young person allocated to the comparison arm to all or part of the intervention and the masking of young people and RAs to intervention status to minimise response and selection bias [26]. Our trial also had a lower than predicted (30% vs 40%) 12 month attrition rate and higher response rates at three and 12 months compared to the only other general practice trial [15]. Participation and attrition rates were balanced between arms, with similar reasons for withdrawal, thus the likelihood of bias in the estimated intervention effects for the risky behaviours was low. This was further supported with the results from the multiple imputation analyses that corrected for any bias in the intervention effect under the assumption that data were missing at random given the observed covariates.

The strength of the trial in examining real world effects of a recommended practice to screen and intervene with young people across multiple health risks is also one of its greatest limitations, in that there are multiple primary outcomes carrying a higher risk of significant findings by chance alone [27]. However, in keeping with other complex interventions, a single primary outcome would have been inadequate to capture the multifaceted nature of the intervention [23, 52]. Arguments for statistical adjustment in the case of multiple outcomes assume that each outcome occurs independently of the other outcomes [52, 53]. However, this is not the case in our study because health risk behaviours usually co-occur in young people [6] and clinicians may also discuss more than one health risk during a consultation. Opponents of statistical adjustment in this scenario highlight the risks of negating potential effects [53]. With the exception of road safety and emotional distress, the risk differences for other risky behaviours were in the same direction indicating that an intervention effect is possible, albeit small, across the individual risk factors. However, our trial was underpowered to detect the small risk reductions observed in the more prevalent tobacco and alcohol use risk behaviours and to detect reductions in the degree of risk taking; we could only examine whether a young person reported engaging in the behaviour or not. Illicit drug use and unprotected sex were less prevalent risks thus the intervention effect was estimated with greater precision and there was more power to detect smaller risk reductions. We chose two time points to measure the effect of the intervention, at three and 12 months to capture immediate and longer term effects, but this also increased the number of outcomes. It is unclear why the changes in sexual health risks were not consistent from three to 12 months. In all, the impact of our intervention on health outcomes is minimal, or in light of the multiple outcomes, inconclusive, with only illicit drug use and risk for unplanned pregnancy being reduced at 12 months (Table 5). The only other trial in general practice that tested a universal intervention for all 16 year olds attending the practice also showed minimal impact on health outcomes with concerns about study power and large drop out over 12 months [15]. It has been suggested that sample sizes necessary to demonstrate whether office-based clinical preventive screening and counselling of young people would be effective and efficient found that these could be prohibitive, and instead recommended these services are implemented based on best-practice knowledge and efficacy studied only as part of widespread implementation [12].

There was potential for selection bias in the post-randomisation sample of young people because clinicians in both arms were not masked to the intervention status of the practice and were not systematically approaching all young people they consulted. This raises the possibility that intervention clinicians may have biased selection by approaching youth they had screened differently to the comparison. However, we believe that the youth recruited in the two study arms are comparable. First, the proportion of young people engaging in risk taking behaviours at exit interview was similar in both arms of the trial for both the pre-randomisation and post-randomisation samples, indicating that differential selection bias between the two study arms was unlikely in our study. There was also no differential non-response across both arms of the trial. Secondly, intervention clinicians received more help from RAs who were masked to the study arm status and were systematic in their approach to all eligible patients attending the study clinicians whereas comparison clinicians, who also had training on health risk screening, recruited more patients unassisted and had more potential to select participants with more risk taking behaviours that they had screened. If this was the case, the intervention effect on whether the clinician discussed the risky behaviours with the young person would be diluted. Thirdly, over 40% of those approached by clinicians in both arms did not consent to participate when recruited by the CATI RA, who was masked to the study allocation, which suggests that any significant selection bias by the clinician is unlikely. Finally, we adjusted for recruitment method in all analyses. Imbalance in the number of young people recruited in each arm may be indicative of a selection bias. However, this imbalance is more likely to be due to the different number of clinicians that recruited in each study arm (53 in intervention and 79 in comparison). This is further supported in that the average number of patients recruited per clinician in each study arm was similar (7.1 intervention and 6.6 comparison). The difference in the number of clinicians between the arms may be partly explained by the two fewer intervention arm practices however it is more likely that the imbalance occurred by chance as practices were randomly allocated to study arms after clinicians had agreed to take part and had completed their baseline assessments.

Most participating clinicians had an interest in young people’s health, as reflected in most having past training, which restricts the generalisability of our findings to such practices. Our earlier work, using similar techniques to train clinicians, also demonstrated shifts in clinician behaviour in an era (1996) when very few had prior training in adolescent health [17]. This suggests that the current intervention could be used in other settings with less primed clinicians. Patients in Australia are not confined to one practice and can choose where to attend; we anticipate practices in real life are therefore more likely to select training based on the needs of their patient population. The number of practices recruited from those expressing interest is typical of participation rates in other Victorian general practice cluster randomised trials [54, 55]. The minimal training offered to comparison clinicians may have served as a refresher, given most had prior training, and enhanced their practice with young people, hence attenuating the intervention effect. We are unable to report on demographic characteristics of young people who were eligible but declined to provide contact details to researchers.

A further limitation was the young people’s self-reported measures on clinician discussion and on risk taking. In determining clinician detection of health risks, based on the concordance between young people reporting they are engaging in the risk behaviour and that they discussed this with the clinician, we have assumed that the young person disclosed the risk during that discussion and that the approaches the clinician used in this discussion were based on a motivational interviewing style, but we cannot know this for certain. In addition there may be recall bias where the young person in either arm may have under-reported discussions that actually took place or may not have reported their own risks during the exit interview. The proportion of young people using drugs detected by clinicians was similar in both arms (Table 3) yet intervention clinicians discussed illicit drugs in nearly twice as many consultations as comparison clinicians (15%, 55/377 vs. 8% 40/524; S2 Table). Given that fewer intervention youth compared to comparison youth reported using illicit drugs at three and 12 months post-consultation, it is possible that some young people did not report their drug use at the exit interview; an alternative possibility is that broader ranging discussion of psychosocial issues is helpful in reducing this risk. Even acknowledging these limitations, the exit interview has been shown to be a valid method of capturing discussions of health risk up to two months after the consultation [56].

The absolute risk reduction observed in illicit drug use remained robust even in the imputed data, suggesting that the effect observed is not because those lost to follow-up were more likely to be using drugs. This difference in drug use by arm, while small, could be considered clinically important in the context that most young people access a primary care clinician at least annually which suggests there is potential to make a large difference at a population level if every visit included a preventive approach. Longer follow-up time would be necessary to test for any cumulative effects on individuals’ behaviours after successive visits to the practice with exposure to repeated discussions about health.

Our intervention had no impact on road safety behaviours. We did not have a validated tool to measure road risk so our study-designed measures for a number of road risks may have been insensitive to change. Other clinical intervention studies demonstrate improvements in helmet wearing by cyclists [51, 57] but have not examined effects on risky driving; this area requires further research.

The lack of impact of the intervention on emotional distress may reflect that management of mental health issues was not a specific focus of the intervention. An effective intervention might have included specific training on evidence based management of depression in young people such as cognitive behavioural therapy [58]. The use of diagnostic instruments for disorder may also be preferable to detect change in mental health status rather than the screening test used in our study.

In the US where guidelines for adolescent preventive services have been in place for the past two decades, adoption of screening by clinicians remains suboptimal especially for mental health risk, which occurs in around 30% of adolescent well care visits [59]. In our study, adoption of the study screening tool, designed to make screening and discussion of risk more systematic and time efficient, was low and overall rates of discussion of each health risk were low. Research on acceptable technologies hold promise for enabling time efficient, systematic screening that enables clinicians to target their interventions, however, methods for sustained and routine implementation of these in practice and the impact of these technologies on health outcomes still require testing [60]. Other studies have used system change interventions over 12 months, in contrast to our brief three-month timeframe, for training and two practice visits, to successfully embed preventive care models for other conditions such as Chlamydia screening, in primary care practices [51, 61]. General practice annual preventive health assessments for other age groups in Australia (e.g. four year olds, 45–49 year olds and over 75 year olds) are funded through national health care, which at least removes financial barriers to the longer consultations required for risk screening [62]. However systematic evaluations of the effect of these activities have been lacking [63].

In the global drive toward universal health coverage, adolescents remain the most neglected age group [64]. One reason has been that adolescents often do not bring their major health needs to clinical encounters. Testing the effectiveness of a complex intervention addressing multiple health issues in the complex setting of primary care where young people are screened opportunistically is challenging, yet these holistic approaches are necessary to address hidden health needs. Our trial has provided inconclusive evidence for improved health outcomes, however it does show that a high proportion of young people attending primary care services are engaging in health risks and that with an intervention there are shifts in clinician behaviour and promising indicators that shifts in young people’s risk taking are possible. Our intervention would be strengthened by technology enabling efficient, engaging and systematic health risk screening and detection, so providers could target counselling toward higher risk individuals, and by adding specific components for managing emotional distress. Our economic evaluation is pending. Larger trials would be needed to replicate our findings and to substantiate the smaller reductions we observed in risk taking for the more prevalent tobacco and alcohol use. Ideally future research would identify a single outcome depicting global health risk. We believe our results lend support for accepted best practice in consulting with adolescents [8, 9] and for the potential of preventatively oriented primary care to better meet the health needs of young people.

## Panel: What This Paper Adds

### What is already known on this topic

- Behavioural counselling across a range of health risks, targeted to young people with specific health risks attending specialist primary care services (antenatal or HIV clinics) have been shown to lower health risks.
- Studies of training and system interventions for primary care clinicians have been shown to improve preventive screening practices during well care visits and in simulated consultations.
- However no studies evaluate the combination of screening for a range of psychosocial health risks and counselling delivered by family doctors and nurses in real world general practice settings, for any and all youth attending general practice for any reason.

### What this study adds

- This trial provides evidence around the potential of primary care to respond to the health needs of young people through screening and counselling across a range of prevalent health risks, during typical consultations, in real world general practice, however impact on health outcomes is inconclusive.
- Larger trials are required to detect reductions in the more prevalent health risk behaviours such as tobacco and alcohol use and longer follow up times will detect any cumulative effects of repeated health discussions delivered in primary care over time.
- The intervention would be strengthened by technology which enables efficient, preventive screening and counselling of youth at risk more systematic and routine work in general practice. Interventions also need to include specific training in responses to emotional distress alongside responding to health risk behaviours.

## Supporting Information

S1 CONSORT Checklist CONSORT CHECKLIST. This file contains a completed CONSORT CHECKLIST for the trial presented in this manuscript.
(DOCX)

S1 File Outline of Study Phases. This file shows a diagram of the main study phases with their accompanying dates.
(PDF)

S2 File Detail of Study Intervention. This file describes the detail of the intervention delivered to practices allocated to the intervention arm.
(DOCX)

S3 File Study measures. This file lists the general, primary and secondary outcome measures used in this study.
(DOCX)

S4 File Definition of psychosocial health risks. This file describes the way we have combined study measures to define psychosocial risks as binary outcomes; that is, whether the risk is present (‘Risk’) or low or absent (‘No Risk’).
(DOCX)

S5 File Missing data and assumptions. This file describes the characteristics of young people participants who were lost to follow-up during the study and how they differ from those remaining in the study for both arms of the trial. It also details our missing data assumptions and our approach to missing data at three and 12 months using multiple imputation.
(DOCX)

S6 File Detail on Secondary Outcomes analysis. This file provides detail on the results of the secondary outcome analyses on Likelihood of returning to the clinician for further visits; trust in the clinician and whether young people would recommend their clinician to a friend.
(DOCX)

S1 Protocol Trial protocol. (PDF)

S1 Table Number of health risks discussed by clinicians with young people at their last visit by study arm. The Table summarises the number of health risks out of the six (tobacco, alcohol and illicit drug use, risk for unplanned pregnancy, risk of STIs and road risks) reported by the young people as having been discussed with their clinician at the last visit by study arm; Sixty percent (222/372) of clinicians in the intervention arm discussed at least one risk factor compared to the 53% (272/516) in the comparison arm. The number of risky behaviours discussed at one consultation was higher in the intervention arm than in the comparison arm.
(DOCX)

S2 Table Clinicians who raised or discussed the health issue with the young person at the consultation. The Table shows clinicians’ likelihood of discussing the six health risk behaviours, emotional distress and fear and abuse in relationships with young people. Intervention clinicians were more likely to discuss tobacco, alcohol and illicit drug use, road risks, and fear and abuse in relationships. There was no evidence to support differences between arms in discussion of sexual health risks and emotional distress.
(DOCX)

## References

[1] Patel V, Ramasundarahettige C, Vijayakumar L, Thakur JS, Gajalakshmi V, Gururaj G, et al Suicide mortality in India: a nationally representative survey. The Lancet. 2012;379(9834):2343–51. 10.1016/S0140-6736(12)60606-0 PMC4247159 22726517

[2] Patton GC, Coffey C, Cappa C, Currie D, Riley L, Gore F, et al Health of the world's adolescents: a synthesis of internationally comparable data. Lancet. 2012;379(9826):1665–75. Epub 2012/04/28. 10.1016/S0140-6736(12)60203-7 22538181

[3] Sawyer SM, Afifi RA, Bearinger LH, Blakemore SJ, Dick B, Ezeh AC, et al Adolescence: a foundation for future health. Lancet. 2012;379(9826):1630–40. Epub 2012/04/28. 10.1016/S0140-6736(12)60072-5 22538178

[4] Viner RM, Coffey C, Mathers C, Bloem P, Costello A, Santelli J, et al 50-year mortality trends in children and young people: a study of 50 low-income, middle-income, and high-income countries. Lancet. 2011;377(9772):1162–74. Epub 2011/04/01. 10.1016/S0140-6736(11)60106-2 21450338

[5] Tylee A, Haller DM, Graham T, Churchill R, Sanci LA. Youth-friendly primary-care services: how are we doing and what more needs to be done? Lancet. 2007;369(9572):1565–73. 17482988 10.1016/S0140-6736(07)60371-7

[6] Coups EJ, Gaba A, Orleans CT. Physician screening for multiple behavioral health risk factors. Am J Prev Med. 2004;27(2 Suppl):34–41. Epub 2004/07/28. 15275672 10.1016/j.amepre.2004.04.021

[7] Klein JD, Matos Auerbach M. Improving adolescent health outcomes. Minerva Pediatrica. 2002;54:25–39. 11862164

[8] American Academy of Pediatrics. Bright Futures: Prevention and health promotion for infants, children, adolescents and their families. Available: http://brightfutures.aap.org/; 2010. Accessed 10 Jan 2010.

[9] Department of Health (UK). Quality criteria for young people friendly health services—2011 edition London: Deaprtment of Health; 2011.

[10] Moyer VA, Butler M. Gaps in the evidence for well-child care: a challenge to our profession. Pediatrics. 2004;114(6):1511–21. Epub 2004/12/03. 15574609 10.1542/peds.2004-1076

[11] Sanci L. Clinical preventive services for adolescents: facing the challenge of proving "an ounce of prevention is worth a pound of cure". J Adolesc Health. 2011;49(5):450–2. Epub 2011/10/25. 10.1016/j.jadohealth.2011.09.002 22018557

[12] Downs SM, Klein JD. Clinical preventive services efficacy and adolescents' risky behaviors. Arch Pediatr Adolesc Med. 1995;149(4):374–9. 7704164 10.1001/archpedi.1995.02170160028004

[13] Chen X, Murphy DA, Naar-King S, Parsons JT. A clinic-based motivational intervention improves condom use among subgroups of youth living with HIV. J Adolesc Health. 2011;49(2):193–8. Epub 2011/07/26. 10.1016/j.jadohealth.2010.11.252 21783053 PMC3282587

[14] Joseph JG, El-Mohandes AA, Kiely M, El-Khorazaty MN, Gantz MG, Johnson AA, et al Reducing psychosocial and behavioral pregnancy risk factors: results of a randomized clinical trial among high-risk pregnant african american women. Am J Public Health. 2009;99(6):1053–61. Epub 2009/04/18. 10.2105/AJPH.2007.131425 19372532 PMC2679805

[15] Walker Z, Townsend J, Oakley L, Donovan C, Smith H, Hurst Z, et al Health promotion for adolescents in primary care: randomized controlled trial. British Medical Journal. 2002;325:524–30. 12217993 10.1136/bmj.325.7363.524 PMC121334

[16] Borowsky IW, Mozayeny S, Ireland M. Brief psychosocial screening at health supervision and acute care visits. Pediatrics. 2003;112(1):129–40. 12837878 10.1542/peds.112.1.129

[17] Sanci LA, Coffey CMM, Veit FCM, Carr-Gregg M, Patton GP, Day N, et al Evaluation of the effectiveness of an educational intervention for general practitioners in adolescent health care: randomised controlled trial. British Medical Journal. 2000;320:224–30. 10642233 10.1136/bmj.320.7229.224 PMC27271

[18] Zwarenstein M, Treweek S, Gagnier JJ, Altman DG, Tunis S, Haynes B, et al Improving the reporting of pragmatic trials: an extension of the CONSORT statement. BMJ. 2008;337:a2390 Epub 2008/11/13. 10.1136/bmj.a2390 19001484 PMC3266844

[19] Lustig JL, Ozer EM, Adams SH, Wibbelsman CJ, Fuster CD, Bonar RW, et al Improving the delivery of adolescent clinical preventive services through skills-based training. Pediatrics. 2001;107(5):1100–7. 11331693 10.1542/peds.107.5.1100

[20] Ozer EM, Adams SH, Lustig JL, Gee S, Garber AK, Gardner LR, et al Increasing the screening and counseling of adolescents for risky health behaviors: a primary care intervention. Pediatrics. 2005;115(4):960–8. 15805371 10.1542/peds.2004-0520

[21] Haines A, Donald A. Introduction In: Haines A, Donald A, editors. Getting research findings into practice. 2nd ed. London: BMJ Publishing Group; 2002 p. 1–10.

[22] Lally P, van Jaarsveld CHM, Potts HWW, Wardle J. How are habits formed: Modelling habit formation in the real world. European journal of social psychology. 2010;40(6):998–1009.

[23] Craig P, Dieppe P, Macintyre S, Michie S, Nazareth I, Petticrew M. Developing and evaluating complex interventions: the new Medical Research Council guidance. BMJ. 2008;337:a1655 Epub 2008/10/01. 10.1136/bmj.a1655 18824488 PMC2769032

[24] Shiell A, Hawe P, Gold L. Complex interventions or complex systems? Implications for health economic evaluation. BMJ. 2008;336(7656):1281–3. 10.1136/bmj.39569.510521.AD 18535071 PMC2413333

[25] Sanci L, Grabsch B, Chondros P, Shiell A, Pirkis J, Sawyer S, et al The prevention access and risk taking in young people (PARTY) project protocol: a cluster randomised controlled trial of health risk screening and motivational interviewing for young people presenting to general practice. BMC Public Health. 2012;12:400 Epub 2012/06/08. 10.1186/1471-2458-12-400 22672481 PMC3533834

[26] Campbell MK, Piaggio G, Elbourne DR, Altman DG. Consort 2010 statement: extension to cluster randomised trials. BMJ. 2012;345:e5661 Epub 2012/09/07. 10.1136/bmj.e5661 22951546

[27] Schulz KF, Altman DG, Moher D. CONSORT 2010 Statement: Updated Guidelines for Reporting Parallel Group Randomized Trials. Annals of Internal Medicine. 2010;152(11):726–32. 10.7326/0003-4819-152-11-201006010-00232 20335313

[28] Ford CA, Millstein SG, Halpern-Felsher BL, Irwin CE Jr. Influence of physician confidentiality assurances on adolescents' willingness to disclose information and seek future health care. JAMA. 1997;278(12):1029–34. 9307357

[29] Thom DH, Campbell B. Patient-physician trust: an exploratory study. Journal of Family Practice. 1997;44(2):169–76. 9040520

[30] Primary Health Care Research & Information Service. Key Division of General Practice characteristics 2009–2010. 2013 [12/03/2014]; Available: http://www.phcris.org.au/products/asd/keycharacteristic/KeyDGPstatistics.xls.

[31] Kessler RC, Barker PR, Colpe LJ, Epstein JF, Gfroerer JC, Hiripi E, et al Screening for serious mental illness in the general population. Arch Gen Psychiatry. 2003;60(2):184–9. Epub 2003/02/13. 12578436 10.1001/archpsyc.60.2.184

[32] NHMRC. Australian Guidelines to Reduce Health Risks from Drinking Alcohol. 2009 [16 August 2013]; Available: http://www.nhmrc.gov.au/guidelines/publications/ds10.

[33] Patton GC, Coffey C, Carlin JB, Degenhardt L, Lynskey M, Hall W. Cannabis use and mental health in young people: cohort study. BMJ. 2002;325(7374):1195–8. 12446533 10.1136/bmj.325.7374.1195 PMC135489

[34] Reid SC, Ukoumunne OC, Coffey C, Teesson M, Carlin JB, Patton GC. Problem alcohol use in young Australian adults. Aust N Z J Psychiatry. 2007;41(5):436–41. Epub 2007/04/28. 17464736 10.1080/00048670701264784

[35] StataCorp. Stata Statistical Software: Release 13. College Station, TX: StataCorp LP; 2013.

[36] Australian Bureau of Statistics. SEIFA: Socio-Economic Indexes for Areas. Canberra: ABS, 2006.

[37] Pfaff JJ, Acres JG, McKelvey RS. Training general practitioners to recognise and respond to psychological distress and suicidal ideation in young people. Medical Journal of Australia. 2001;174:222–6. 11280692 10.5694/j.1326-5377.2001.tb143241.x

[38] Eldridge SM, Ashby D, Feder GS, Rudnicka AR, Ukoumunne OC. Lessons for cluster randomized trials in the twenty-first century: a systematic review of trials in primary care. Clinical Trials. 2004;1:80–90. 16281464 10.1191/1740774504cn006rr

[39] Moon L, Meyer P, Grau J. Australia's young people: their health and well-being Canberra: Australian Institute of Health and Welfare; 1999.

[40] World Health Organisation. Mental Illness in General Health Care: An International Study. Ustin T, Sartorius N, editors. West Sussex, UK: John Wiley & Sons Ltd.; 1995.

[41] Kamb ML, Fishbein M, Douglas JM Jr., Rhodes F, Rogers J, Bolan G, et al Efficacy of risk-reduction counseling to prevent human immunodeficiency virus and sexually transmitted diseases: a randomised controlled trial. JAMA. 1998;280(13):1161–7. 9777816 10.1001/jama.280.13.1161

[42] Carne A. Summary Data Report of the 2011–2012 Annual Survey of Divisions of General Practice, Chapter 3: Division Context. Adelaide: 2013.

[43] Primary Health Care Research & Information Service. Female GP numbers by state, 2010–11 2013 [9 January 2014]; Available: http://www.phcris.org.au/fastfacts/fact.php?id=8299.

[44] Britt H, Miller G, Charles J, Henderson J, Bayram C, Valenti L, et al. General practice activity in Australia, 2010–11. Sydney: 2011.

[45] Primary Health Care Research and Information Service. General practice size in Australia, 2005–06 to 2010–11 2013 [9/01/2014]; Available: http://www.phcris.org.au/fastfacts/fact.php?id=4970&search=number+of+solo+practices.

[46] Commonwealth Department of Health and Aged Care. Measuring Remoteness: Accessibility/Remoteness Index of Australia (ARIA) Canberra: ABS, 2001.

[47] Booth ML, Knox S, Kang M. Encounters between adolescents and general practice in Australia. J Paediatr Child Health. 2008;44(12):699–705. Epub 2008/12/17. 10.1111/j.1440-1754.2008.01409.x 19077068

[48] Family Medicine Research Centre. ICPC-2 Plus Demonstrator. University of Sydney; 1998 [10/05/2013]; Available: http://sydney.edu.au/medicine/fmrc/icpc-2-plus/demonstrator/icpc-demo.php?_PROGRAM=sascode.icpcdemo_in.sas.

[49] Goldenring JM, Rosen DS. Getting into adolescent heads: An essential update. Contemporary Pediatrics. 2004;21:64–90.

[50] Bradford S, Rickwood D. Psychosocial assessments for young people: a systematic review examining acceptability, disclosure and engagement, and predictive utility. Adolescent Health, Medicine and Therapeutics. 2012:111. 10.2147/AHMT.S38442 PMC3915791 24600292

[51] Ozer EM, Adams SH, Orrell-Valente JK, Wibbelsman CJ, Lustig JL, Millstein SG, et al Does delivering preventive services in primary care reduce adolescent risky behavior? J Adolesc Health. 2011;49(5):476–82. Epub 2011/10/25. 10.1016/j.jadohealth.2011.02.011 22018561

[52] Goodman SN. Multiple Comparisons, Explained. Am J Epidemiol. 1998;147(9):807–12. 9583709 10.1093/oxfordjournals.aje.a009531

[53] Rothman KJ. No Adjustments Are Needed for Multiple Comparisons. Epidemiology. 1990;1(1):43–6. 2081237

[54] Blackberry ID, Furler JS, Best JD, Chondros P, Vale M, Walker C, et al Effectiveness of general practice based, practice nurse led telephone coaching on glycaemic control of type 2 diabetes: the Patient Engagement and Coaching for Health (PEACH) pragmatic cluster randomised controlled trial. BMJ. 2013;347:f5272 Epub 2013/09/21. 10.1136/bmj.f5272 24048296 PMC3776648

[55] Hegarty K, O'Doherty L, Taft A, Chondros P, Brown S, Valpied J, et al Screening and counselling in the primary care setting for women who have experienced intimate partner violence (WEAVE): a cluster randomised controlled trial. Lancet. 2013;382(9888):249–58. Epub 2013/04/20. 10.1016/S0140-6736(13)60052-5 23598181

[56] Klein JD, Graff CA, Santelli JS, Hedberg VA, Allan MJ, Elster AB. Developing quality measures for adolescent care: validity of adolescents' self-reported receipt of preventive services. Health Serv Res. 1999;34(1 Pt 2):391–404. Epub 13/04/1999. 10199683 PMC1089009

[57] Stevens MM, Olson AL, Gaffney CA, Tosteson TD, Mott LA, Starr P. A Pediatric, Practice-Based, Randomized Trial of Drinking and Smoking Prevention and Bicycle Helmet, Gun, and Seatbelt Safety Promotion. Pediatrics. 2002;109(3):490–7. 11875146 10.1542/peds.109.3.490

[58] National Collaborating Centre for Mental Health. Depression in Children and Young People. Identification and management in primary, community and secondary care Manchester, U.K.: National Institute for Health and Clinical Excellence, 2005.

[59] Ozer EM, Zahnd EG, Adams SH, Husting SR, Wibbelsman CJ, Norman KP, et al Are adolescents being screened for emotional distress in primary care? J Adolesc Health. 2009;44(6):520–7. 10.1016/j.jadohealth.2008.12.016 19465315

[60] Olson AL, Gaffney CA, Hedberg VA, Gladstone GR. Use of Inexpensive Technology to Enhance Adolescent Health Screening and Counseling. Arch Pediatr Adolesc Med. 2009;163(2):172–7. 10.1001/archpediatrics.2008.533 19188650

[61] Margolis PA, Lannon CM, Stuart JM, Fried BJ, Keyes-Elstein L, Moore DE Jr. Practice based education to improve delivery systems for prevention in primary care: randomized trial. British Medical Journal. 2004;328:388–98. 14766718 10.1136/bmj.38009.706319.47 PMC341391

[62] Australian Government Department of Health and Ageing. Fact sheeets and proformas on Medicare health assessment items. 2014 [cited 2014]; Available: http://www.commcarelink.health.gov.au/internet/main/publishing.nsf/Content/mha_resource_kit.

[63] Beilby JJ. Primary care reform using a layered approach to the Medicare Benefits Scheme: unpredictable and unmeasured. Med J Aust. 2007;187(2):69–71. Epub 2007/07/20. 17635084 10.5694/j.1326-5377.2007.tb01141.x

[64] World Health Organisation. Universal health coverage—a new framework for action. [17/06/2014]; Available: http://apps.who.int/adolescent/second-decade/section6/page1/universal-health-coverage.html.

