# Supplementary figures and images for: Responding to Young People’s Health Risks in Primary Care: A Cluster Randomised Trial of Training Clinicians in Screening and Motivational Interviewing
Source: PLoS One. 2015 Sep 30;10(9):e0137581. doi: 10.1371/journal.pone.0137581 (PMC4589315; doi:10.1371/journal.pone.0137581)

## S1 File. Outline of study phases

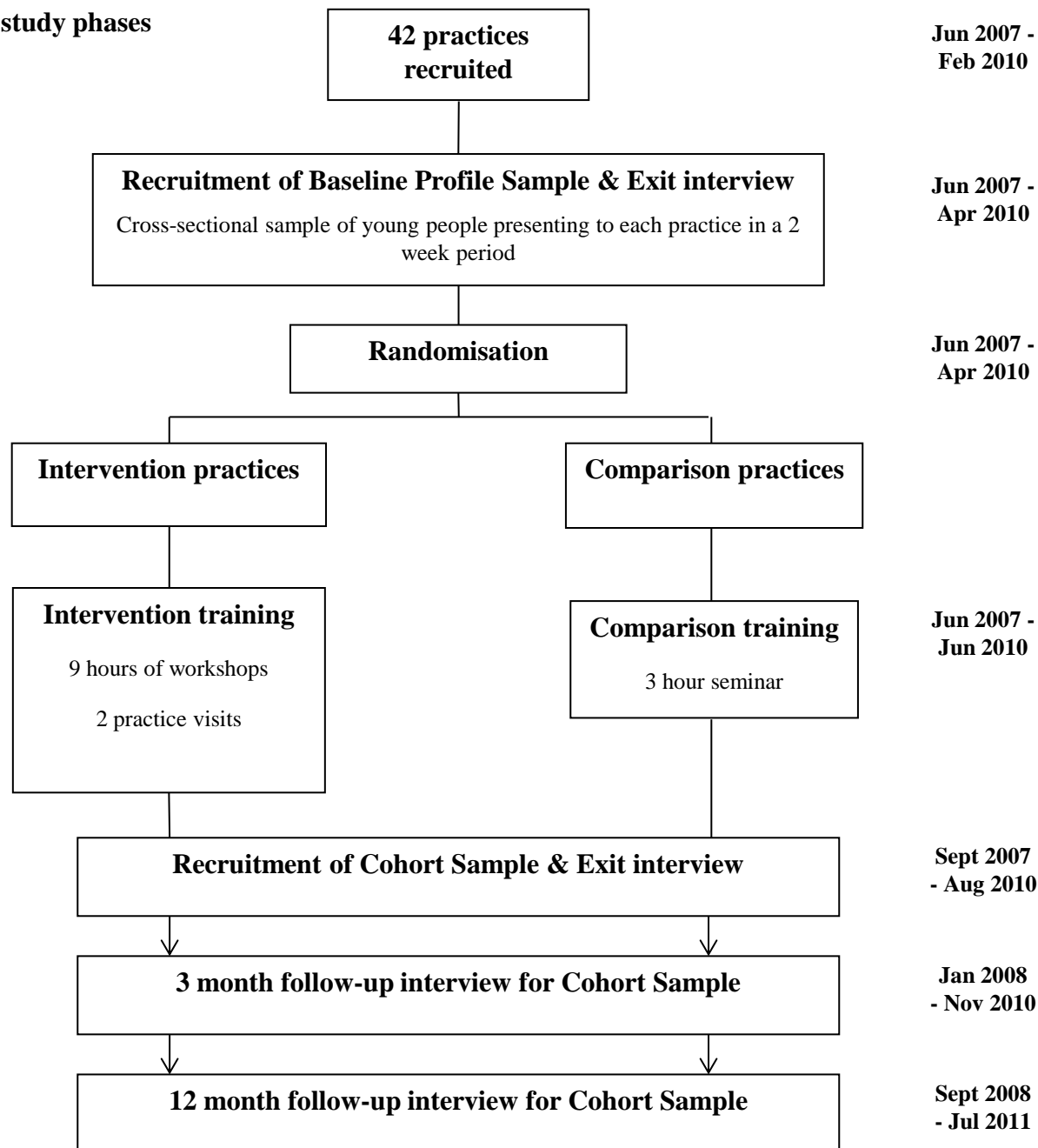

Supplement: S1 File — This file shows a diagram of the main study phases with their accompanying dates. (PDF) [file pone.0137581.s002.pdf]
