# Supplementary material for: Responding to Young People’s Health Risks in Primary Care: A Cluster Randomised Trial of Training Clinicians in Screening and Motivational Interviewing
Source: PLoS One. 2015 Sep 30;10(9):e0137581. doi: 10.1371/journal.pone.0137581 (PMC4589315; doi:10.1371/journal.pone.0137581)
Supplement: S2 File — This file describes the detail of the intervention delivered to practices allocated to the intervention arm. (DOCX) [file pone.0137581.s003.docx]

**S2 File. Detail of Study Intervention.**

Workshops covered three topics of three hours each: youth-friendly care; screening for and discussing health risks using the HEADSS framework [1]; and providing a response to detected risky behaviours with a brief intervention based on motivational interviewing principles, including health promotion advice [2]. Adolescent actors allowed clinicians to practice new skills by role play and provided feedback and coaching in youth-friendly communication skills [3, 4]. Two to three hours of interactive training in youth-friendly care was also provided to practice support staff (PSS: receptionists and practice managers). Training was delivered to each practice by an expert in adolescent primary care (LS) either at the practice or a local venue. During workshops, clinicians were introduced to the study screening tool designed to prompt them to raise and discuss health risk behaviours and also protective factors and strengths with their patients [2].

After the workshops, two practice visits were conducted by LS and a research assistant (RA), two weeks apart. Using the plan-do-study-act (PDSA) cycle of continuous quality improvement [5], practices were assisted with integrating screening into office and clinical procedures. The RA also assisted with updating practice referral lists with local youth specialist services, and provided posters and pamphlets addressing youth-friendly care (e.g. confidentiality) and health risk behaviours (e.g. road safety). Data collected from the profile exit interviews (Fig. 1) were presented to participating clinicians and PSS to help them identify aspects of care that could be improved. These data included patients’ risk profile, whether clinicians discussed health behaviours during the consultation, and the young person’s satisfaction, trust [6], and likelihood to return to the practice [7].

# References

1. Goldenring JM, Rosen DS. Getting into adolescent heads: An essential update. Contemporary Pediatrics. 2004;21:64-90.

2. Sanci L, Grabsch B, Chondros P, Shiell A, Pirkis J, Sawyer S, et al. The prevention access and risk taking in young people (PARTY) project protocol: a cluster randomised controlled trial of health risk screening and motivational interviewing for young people presenting to general practice. BMC Public Health. 2012;12:400. Epub 2012/06/08.

3. Cahill HW. Learning partnerships : the use of poststructuralist drama techniques to improve communication between teachers, doctors and adolescents [Doctorate]. Melbourne: University of Melbourne; 2008.

4. Sanci LA, Coffey CMM, Veit FCM, Carr-Gregg M, Patton GP, Day N, et al. Evaluation of the effectiveness of an educational intervention for general practitioners in adolescent health care: randomised controlled trial. British Medical Journal. 2000;320:224-30.

5. Langley GK, Nolan T, Nolan C, Norman PL. The improvement guide: a practical approach to enhancing organizational performance. San Francisco: Jossey-Bass; 1996.

6. Thom DH, Campbell B. Patient-physician trust: an exploratory study. Journal of Family Practice. 1997;44(2):169-76.

7. Ford CA, Millstein SG, Halpern-Felsher BL, Irwin CE, Jr. Influence of physician confidentiality assurances on adolescents' willingness to disclose information and seek future health care. JAMA. 1997;278(12):1029-34.
