# Supplementary material for: Responding to Young People’s Health Risks in Primary Care: A Cluster Randomised Trial of Training Clinicians in Screening and Motivational Interviewing
Source: PLoS One. 2015 Sep 30;10(9):e0137581. doi: 10.1371/journal.pone.0137581 (PMC4589315; doi:10.1371/journal.pone.0137581)
Supplement: S3 File — This file lists the general, primary and secondary outcome measures used in this study. (DOCX) [file pone.0137581.s004.docx]

**S3 File. Study Measures**

**General measures:**

- Demographic details were collected about the practice (size, location, socioeconomic index based on postcode [1] and billing status), the clinicians (age, gender, country and year of graduation and prior training received in adolescent health), and the young people (age, gender, country of birth, Aboriginal or Torres Strait Islander status, education and employment status, reason for presentation to the clinic and whether accompanied by a parent or guardian, and frequency of consulting a practice clinician or any other practice).
- Research staff documented whether young people were recruited by the clinician or research assistant.

**Primary outcome measures:**

- Clinician detection of health risks was defined as clinician’s detection of at least one of the six health risk behaviors in the young person (ie tobacco, alcohol or illicit drug use, sexually transmitted infection, unplanned pregnancy and road safety risks). Clinician detection of each health risk behavior was determined when both a discussion of the health risk had taken place with the clinician at that consultation (yes/no), as reported by the young person in the cohort exit interview, and whether the young person also reported engaging in that health risk (yes/no). True ‘clinician accuracy of detection’ (comparing self-reported health risk behaviours in the cohort exit interview with clinician diagnosis in encounter forms) could not be estimated as clinicians did not routinely record a diagnosis about the presence of specific health risk behaviours and they could not be cued in the encounter form to do so without this becoming an intervention in itself.

Self-reported health risks measured in the CATI interviews included: use of tobacco, alcohol and other substances in the past month and 12 months [2, 3], safe sex practices (against sexually transmitted infection and unplanned pregnancy), and road safety (do not use of helmets when riding a bicycle or motor bike, do not wear seatbelt as a driver, do not wear seatbelt use as a passenger, speeding more than 10km/hour over the limit, use of handheld mobile phones while driving, travelling with people under the influence of alcohol or drugs, and driving under the influence of alcohol or drugs) using a categorical response set of ‘never’, ‘rarely’, ‘sometimes’, ‘most of the time’ and ‘always’, with an additional option of ‘not applicable’.

**Secondary outcome measures** in the exit interview were validated measures on the young person’s:

- Likelihood of return for future visits [4]
- Trust in the family doctor [5]
- Emotional distress was measured with the Kessler Psychological Distress Scale (K10) [6]
- Fear and Abuse in relationships was measured only in young people aged 17 years or older using the screening questions of the composite abuse scale [7] and was positive if the participant answered ‘yes’ to ever having been afraid of a partner, or a family member, or was ever forced to have sex.

Clinician detection of emotional distress or abuse (as above) was classified as positive when there was concordance in young people’s reports of both having discussed these topics with their clinician and reporting that they had these health issues.

Acceptability of clinicians’ use of a psychosocial health risk screening tool to identify risk and prompt discussion was assessed by asking young people at the “exit interview” whether they thought completing a ‘lifestyle questionnaire’ was a ‘good idea’, ‘bad idea’ or ‘unsure’. The frequencies of response in each category were described for the intervention arm participants only.

**References**

1. Australian Bureau of Statistics. SEIFA: Socio-Economic Indexes for Areas. Canberra: ABS, 2006.

2. RISQ. DEP-ADO: Detection of Alchohol and Drug Problems in Adolescents - Version 3.2. 2007; Available: http://www.risqtoxico.ca/risq/www/dep_ado.php.

3. Sanci L, Grabsch B, Chondros P, Shiell A, Pirkis J, Sawyer S, et al. The prevention access and risk taking in young people (PARTY) project protocol: a cluster randomised controlled trial of health risk screening and motivational interviewing for young people presenting to general practice. BMC Public Health. 2012;12:400. Epub 2012/06/08.

4. Ford CA, Millstein SG, Halpern-Felsher BL, Irwin CE, Jr. Influence of physician confidentiality assurances on adolescents' willingness to disclose information and seek future health care. JAMA. 1997;278(12):1029-34.

5. Thom DH, Campbell B. Patient-physician trust: an exploratory study. Journal of Family Practice. 1997;44(2):169-76.

6. Kessler RC, Barker PR, Colpe LJ, Epstein JF, Gfroerer JC, Hiripi E, et al. Screening for serious mental illness in the general population. Arch Gen Psychiatry. 2003;60(2):184-9. Epub 2003/02/13.

7. Hegarty K, Bush R, Sheehan M. The composite abuse scale: further development and assessment of reliability and validity of a multidimensional partner abuse measure in clinical settings. Violence and victims. 2005;20(5):529-47. Epub 2005/10/27.
