# Supplementary material for: Responding to Young People’s Health Risks in Primary Care: A Cluster Randomised Trial of Training Clinicians in Screening and Motivational Interviewing
Source: PLoS One. 2015 Sep 30;10(9):e0137581. doi: 10.1371/journal.pone.0137581 (PMC4589315; doi:10.1371/journal.pone.0137581)
Supplement: S4 File — This file describes the way we have combined study measures to define psychosocial risks as binary outcomes; that is, whether the risk is present (‘Risk’) or low or absent (‘No Risk’). (DOCX) [file pone.0137581.s005.docx]

| **S4 File. Definition of psychosocial health risks** | | | | |  |
| --- | --- | --- | --- | --- | --- |
|  |  | |  | | |
| **Clinical Level of risk** | | **Algorithm for determining risk** | | **Definition of binary outcome Risk vs ^#^No Risk for behaviours in Table 4 a & b** | |
| **Emotional Distress** | | | |  | |
| Low | | K10 score less than 20 | | NO RISK | |
| Moderate | | K10 ^s^core between 20 to 24 | | RISK | |
| High | | K10 score between 25 to 29 | |  |  |
| Very high | | K10 score over 29 | |  |  |
| **Tobacco smoking*** | | | |  | |
| No risk | | Not a current smoker, or tried one to two times | | NO RISK | |
| Moderate | | Anything more than tried once or twice; occasional smoking in last 12 months or smoked approximately once per month, weekends or once or twice during the week, 3 times or more a week but not every day | | RISK | |
| High | | **S**mokes everyday | |  |  |
| **Alcohol use** | | | |  | |
| No | | Has never drank alcohol, or not currently a drinker | |  | |
| Low | |  | | NO RISK | |
| Under 16 | | Has the occasional drink in the last 12 months; (only at Exit interview) | |  | |
| 16 or over | | Occasional drink; Once a month; Weekends or once or twice a week; and binge drink less than once a month (at most 12 occasions in past 12 months at the exit interview; at most 3 occasions in past 3 months; at most 1 occasion in past month) | |  | |
| High | |  | |  | |
| Under 16 | | Once a month, weekends or once or twice a week; or any binge drinking | | RISK | |
| 16 or over | | 3 or more times per week or everyday or binge drinking once or more per month  (12 or more occasions for binge drinking times in past 12 months; 4 or more times in past 3 months; 2 or more times in the past month) | |  | |
| **Illicit drug use (including cannabis)** | | | |  | |
| No | | Not currently using or never used | | NO RISK | |
| Low | | Using less than once a week | | RISK | |
| High | | Using once a week or more | |  |  |
| **Road safety** | | | |  | |
| Low | | Never having risky behaviours or always following the safety recommendations (never or not applicable) | | NO RISK | |
| High | | Any risky behaviour or not always following safety recommendations | | RISK | |
| Summary | | Sum of all 7 risk factors and categorise as: 0 "No risk factors" 1 "One risk" 2 "2 or more risks" | |  |  |

**S 2 File. Definition of psychosocial health risks (continued)**

| **STI risk** | |  |
| --- | --- | --- |
| No | Not sexually active |  |
| Low | Sexually active AND 100% condom use, or mostly condom use and less than 3 sexual partners in the last 3 months / trying to get pregnant or in long term relationship (one partner or no partners) | NO RISK |
| High | Sexually active and mostly condom use with 3 or more sexual partners in the last 3 months / sexually active and condom use “sometimes, rarely, never” / trying to get pregnant or in long term relationship (with more than one partner in last 3 months) | RISK |
| **Unplanned pregnancy risk** | |  |
| Low | Not sexually active or sexually active vaginal and trying to get pregnant or in a same sex relationship; Sexually active vaginal and not trying to get pregnant and 100% contraceptive use | NO RISK |
| High | Sexually active vaginal and not trying to get pregnant and less than 100% contraceptive use | RISK |
| **Fear or abuse in relationship^†^** | |  |
| No | Not felt afraid of family members or partners, and not been forced to have sex in last 12 months | NO RISK |
| High | Felt afraid of family members or partners or been forced to have sex in last 12 months | RISK |

Abbreviations: K10=Kessler Psychological Distress Scale [1]; STI=sexually transmitted infection

* Questions were slightly different between exit interview, three and 12 month surveys. Also there were 13 cases at three months and 93 cases at 12 months who smoked but could not be classified as low or high risk, these were coded as missing

# ‘No risk’ includes risk status which may be ‘low’ rather than absent to allow for risk definition as a binary variable

† Only young people 17 years of age or older were asked this question.

**References**

1. Kessler RC, Barker PR, Colpe LJ, Epstein JF, Gfroerer JC, Hiripi E, et al. Screening for serious mental illness in the general population. Arch Gen Psychiatry. 2003;60(2):184-9. Epub 2003/02/13.
