# Supplementary material for: Responding to Young People’s Health Risks in Primary Care: A Cluster Randomised Trial of Training Clinicians in Screening and Motivational Interviewing
Source: PLoS One. 2015 Sep 30;10(9):e0137581. doi: 10.1371/journal.pone.0137581 (PMC4589315; doi:10.1371/journal.pone.0137581)
Supplement: S5 File — This file describes the characteristics of young people participants who were lost to follow-up during the study and how they differ from those remaining in the study for both arms of the trial. It also details our missing data assumptions and our approach to missing data at three and 12 months using multiple imputation. (DOCX) [file pone.0137581.s006.docx]

**S5 File. Missing data and assumptions**

There were nominal differences in the proportion of missing responses between the intervention and comparison arms at three (23% vs 20%) or 12 month (31% in both arms) follow-up or reasons for loss to follow up. There were no missing responses on the covariates fitted in the multivariable models, except when adjusting for the long term health risks measured at the exit interview (responses missing: tobacco smoking<1%, drinking=4%, illicit drug use<1%). Missing responses related to whether the clinician raised or discussed the health issue with the young person was less than 2% in both study arms and in this instance the missing data are unlikely to bias the estimates of the intervention effect. However, a greater proportion of participants had missing responses across the seven items for health risks behaviours measured at 3 (missing responses =21-22%) and 12 (missing responses=31-32%) months.

**Variables associated with missing outcome data at three and 12 months**

Logistic regression using generalised estimating equations with robust standard errors was used to identify variables associated with missing responses at three and 12 months, respectively. In summary, results showed that those with missing data at three (N=188) and 12 (N=281) months were more likely than those remaining in the trial to: be neither studying nor working (13% vs. 5%); have one or more road safety risks (83% vs. 77%) or unplanned pregnancy risks (17% vs. 11%); and lower mean trust in the clinician scores (mean (SD): 74.5(13·7) vs. 76.5(12·5) at 12 months). Being male (29% vs. 23%) and having been recruited by the RA (32% vs. 24%) were more likely in those lost at three months, in addition to having lower mean average on the GPAQ communication score (mean (SD): 78.5 (18.0) vs 81.7 (15.6) at three months) [1, 2]. Having a smoking risk (47% vs. 35%) was more common in those lost at 12 months. Tables of results can be provided on request.

**Multiple imputation approach for missing outcome data at three and 12 months**

Analysis of young people’s health risks presented in Table 4 included all available data (complete cases) and is valid under the restrictive assumption that data are missing completely at random. A multilevel multiple imputation approach using the REALCOM Impute software [3] for health risk behaviours was used to correct for any bias in the complete case analysis and allowed us to relax the missing data assumption to missing at random. A multivariate latent normal model with random effects was used to impute the missing data that respected the multi-level structure of the data, namely, the repeated outcome measures on young people over time (level 1) and individuals clustered within the practice (level 2). The imputation model included health risk factors (smoking, drinking, illicit drug use, unplanned pregnancy, STI, road risks and mental health) measured at exit interview, three and 12 months, variables included in the multivariable model in the main analysis (age, sex and recruitment method of young people, socio-economic status of practice location and billing type) and auxiliary variables collected at the exit interview that were predictive of missing outcomes at three and 12 months (clinician trust scores, General Practice Assessment Questionnaire [GPAQ] communication score [1, 2], and whether the young person was working or not). Continuous variables included in the imputation model were checked for deviations from the normal distribution. GPAQ communication score and trust in clinician score did not follow a normal distribution and were transformed accordingly. The imputation model was fitted using Markov Chain Monte Carlo (MCMC) simulation techniques. Missing data were drawn after 100 burn-in iterations with 4,000 monitored MCMC iterations. The 40 multiply imputed data sets where imported into Stata 13 Statistical software [4] where the main analysis models of interest were fitted to each of the imputed data sets and estimates of the intervention effect were combined using Rubin’s rules [5]. Results of the multiple imputation analyses assuming data are missing at random are presented in Table 5.

Pattern mixture models were also used to assess the robustness of the assumption of the missing data mechanism of each health risk behaviour. The analysis was a logistic regression model for each risk factor at three and 12 months adjusted for patient recruitment by the clinician or RA, patient sex and age and baseline risk factor status where appropriate using available cases. A range of plausible values (0, 0.5, 1, 2) were considered for the informatively missingness odds ratio (IMOR) which is defined as the odds ratio for having a health risk among youth with missing data relative to having a health risk among youth with observed outcome data. In the sensitivity analysis, the IMOR was assumed the same in both arms, fixed at zero in the comparison arm and 0.5, 1 or 2 in the intervention arm and fixed at zero in the intervention arm and 0.5, 1 and 2 in the comparison arm only.

The main analysis presented in Tables 4 and 5 assumed that IMOR was 1 in both study arms. As attrition was similar in the two study arms at three and 12 months follow up, the estimated intervention effect for the risky behaviours remained relatively unbiased under the assumption that data are missing not at random (MNAR) when the IMOR was the same in the two arms (results not shown). Given that the reasons for attrition was similar in both study arms, we could reasonably assume that the health risk profiles of young people with missing data do not differ considerably between the two study arms.

Study conclusions could potentially change if we assumed that youth with missing outcome data in the intervention arm differed in their risky behaviours compared to youth with missing outcome data in the comparison arm. The degree to which the health risk behaviors for youth with missing data varied between the intervention and comparison arms depended on the prevalence of each risky behavior and the untestable assumptions about the risky behaviours of the youth with missing observations in the two study arms. For instance, the adjusted odds ratio for an unplanned pregnancy at 12 months given in Table 4 was 0.4 (95% CI: 0.2 to 0.8). However, in the sensitivity analysis when the IMOR was set to 0.5 in the intervention arm only, the 95% confidence interval for the estimated intervention effect included the odds ratio of 0.4, but also the odds ratio of 1, which was close to the upper bound of the confidence interval. Thus, if young people with a risky behavior in the intervention arm were more likely to be lost to follow up than in the comparison arm, then observed differences favouring the intervention arm will diminish.

When IMOR was set to 0.5 for risk factors such as tobacco use, alcohol use and road safety risks for the comparison arm only, the 95% confidence intervals for the intervention effect excluded odds ratio 1 and the results favoured the intervention. However, when the IMOR was assumed to be around 0.5 for the intervention arm only, the results showed a significant reduction in risky behaviours in the comparison arm compared to the intervention arm. Note that assumptions about different IMORs for the study arms had a greater impact on the estimated intervention effect for tobacco use, alcohol use and road safety risks because the observed prevalence for these risk factors was greater than for illicit drug use, unplanned pregnancy and STIs.

**References**

1. Mead N, Bower P, Roland M. The General Practice Assessment Questionnaire (GPAQ) - development and psychometric characteristics. BMC Fam Pract. 2008;9:13. Epub 2008/02/22.

2. Sanci L, Grabsch B, Chondros P, Shiell A, Pirkis J, Sawyer S, et al. The prevention access and risk taking in young people (PARTY) project protocol: a cluster randomised controlled trial of health risk screening and motivational interviewing for young people presenting to general practice. BMC Public Health. 2012;12:400. Epub 2012/06/08.

3. Carpenter J, Goldstein H, Kenward M. REALCOM-IMPUTE Software for Multilevel Multiple Imputation with Mixed Response Types. Journal of Statistical Software. 2011;45(5):1-14.

4. StataCorp. Stata Statistical Software: Release 13. College Station, TX: StataCorp LP; 2013.

5. Rubin DB. Multiple imputation for nonresponse in surveys. Hoboken: John Wiley & Sons Inc; 1987.
