# Supplementary material for: Responding to Young People’s Health Risks in Primary Care: A Cluster Randomised Trial of Training Clinicians in Screening and Motivational Interviewing
Source: PLoS One. 2015 Sep 30;10(9):e0137581. doi: 10.1371/journal.pone.0137581 (PMC4589315; doi:10.1371/journal.pone.0137581)
Supplement: S6 File — This file provides detail on the results of the secondary outcome analyses on Likelihood of returning to the clinician for further visits; trust in the clinician and whether young people would recommend their clinician to a friend. (DOCX) [file pone.0137581.s007.docx]

**S6 File. Detail on Secondary Outcomes analysis**

Likelihood of returning to the clinician for further visits [[1](#_ENREF_1)] was high, with 97% of young people (347/359 in intervention arm and 482/496 in comparison arm, unadjusted OR 0·87, 95% CI 0·37 to 2·1, p-value=0·76) responding that they will probably or definitely return to the clinician for at least one of these concerns: physical complaints, concern related to eating or exercise, help with a difficult problem, private or sensitive concerns, problems with sex, cigarette smoking, alcohol use, illicit drug use, parents, friends, partner, problems at school, university or work, or an emotional concern including ending life. Young people in both arms also rated trust in their clinician [[2](#_ENREF_2)] as high (intervention mean=74·6, SD=13·6, n=337; comparison mean 76·7, SD=12·3, n=497; unadjusted mean difference=-2·2, 95% CI -5·0 to 0·66, p-value=0·13). Additionally, 93% (800/863) of young people overall would probably or definitely recommend their clinician to a friend.

**References**

1. Ford CA, Millstein SG, Halpern-Felsher BL, Irwin CE, Jr. Influence of physician confidentiality assurances on adolescents' willingness to disclose information and seek future health care. JAMA. 1997;278(12):1029-34.

2. Thom DH, Campbell B. Patient-physician trust: an exploratory study. Journal of Family Practice. 1997;44(2):169-76.
