# Supplementary material for: Responding to Young People’s Health Risks in Primary Care: A Cluster Randomised Trial of Training Clinicians in Screening and Motivational Interviewing
Source: PLoS One. 2015 Sep 30;10(9):e0137581. doi: 10.1371/journal.pone.0137581 (PMC4589315; doi:10.1371/journal.pone.0137581)
Supplement: S1 Table — The Table summarises the number of health risks out of the six (tobacco, alcohol and illicit drug use, risk for unplanned pregnancy, risk of STIs and road risks) reported by the young people as having been discussed with their clinician at the last visit by study arm; Sixty percent (222/372) of clinicians in the intervention arm discussed at least one risk factor compared to the 53% (272/516) in the comparison arm. The number of risky behaviours discussed at one consultation was higher in the intervention arm than in the comparison arm. (DOCX) [file pone.0137581.s009.docx]

**S1 Table. Number of health risks discussed by clinicians with young people at their last visit by study arm in cohort sample of young people**

| **No. of risk discussed** | **Intervention**  **(N=372)** | | **Comparison**  **(N=516)** | |
| --- | --- | --- | --- | --- |
|  | **n** | **(%)** | **n** | **(%)** |
| 0 | 150 | (40.3) | 244 | (47.3) |
| 1 | 56 | (15.1) | 89 | (17.3) |
| 2 | 70 | (18.8) | 103 | (20.0) |
| 3 | 42 | (11.3) | 42 | (8.1) |
| 4 | 30 | (8.1) | 25 | (4.8) |
| 5 | 12 | (3.2) | 12 | (2.3) |
| 6 | 12 | (3.2) | 1 | (0.2) |
