# Supplementary material for: Responding to Young People’s Health Risks in Primary Care: A Cluster Randomised Trial of Training Clinicians in Screening and Motivational Interviewing
Source: PLoS One. 2015 Sep 30;10(9):e0137581. doi: 10.1371/journal.pone.0137581 (PMC4589315; doi:10.1371/journal.pone.0137581)
Supplement: S2 Table — The Table shows clinicians’ likelihood of discussing the six health risk behaviours, emotional distress and fear and abuse in relationships with young people. Intervention clinicians were more likely to discuss tobacco, alcohol and illicit drug use, road risks, and fear and abuse in relationships. There was no evidence to support differences between arms in discussion of sexual health risks and emotional distress. (DOCX) [file pone.0137581.s010.docx]

**S2 Table. Clinicians who raised or discussed the health issue**^*^ **with the young person at the consultation (N=901)**

|  | **Intervention**  **(N=377)** | | | **Comparison**  **(N=524)** | | | **Unadjusted** | | | **Adjusted^§^** | | |
| --- | --- | --- | --- | --- | --- | --- | --- | --- | --- | --- | --- | --- |
| **Psychosocial risks** | **n** | **(%)** | **ICC^†^** | **n** | **(%)** | **ICC^†^** | **OR**^‡^ | **(95% CI)** | ***P*** | **OR**^‡^ | **(95% CI)** | ***P*** |
| **Tobacco use** | 119 | (32·1) | 0·053 | 120 | (23·3) | 0·031 | 1·51 | (0·99 to 2·30) | 0·06 | 1·55 | (1·02 to 2·36) | 0·04 |
| **Alcohol use** | 111 | (29·9) | 0·062 | 85 | (16·5) | 0·046 | 2·07 | (1·27 to 3·39) | 0·004 | 2·18 | (1·32 to 3·60) | 0·002 |
| **Illicit drug use** | 55 | (14·9) | 0·022 | 40 | (7·8) | 0·008 | 2·01 | (1·23 to 3·30) | 0·01 | 1·88 | (1·16 to 3·06) | 0·01 |
| **Sexual health**^**^ | 150 | (40·3) |  | 203 | (39·3) |  |  |  |  |  |  |  |
| Contraception | 139 | (37.5) | 0·083 | 193 | (37.6) | 0·060 | 0·95 | (0·60 to 1·49) | 0·83 | 0·85 | (0·56 to 1·31) | 0·46 |
| Protection from STIs | 112 | (30.2) | 0·062 | 142 | (27.5) | 0·061 | 1·09 | (0·69 to 1.72) | 0·71 | 1·02 | (0.66 to 1·58) | 0·91 |
| **Road and driving safety** | 38 | (10·2) | 0·107 | 7 | (1·4) | 0·002 | 8·43 | (2·77 to 25·6) | 0·0002 | 8·91 | (3·08 to 25·8) | <0·0001 |
| **Emotional distress** | 125 | (33·9) | 0·086 | 149 | (28·9) | 0·043 | 1·25 | (0·79 to 1·97) | 0·34 | 1·31 | (0·86 to 2·02) | 0·21 |
| **Fear or abuse in relationships**^††^ | 23 | (7.2) | 0.054 | 4 | (1·0) | -- | 8.61 | (2.59 to 28.7) | <0·0001 | 9.87 | (3.12 to 31·2) | <0·0001 |

Abbreviations: OR=Odd ratio; CI=Confidence Interval; *P*=P-value; STIs= sexually transmitted infections; Totals vary due to missing responses

Note the 901 young people were recruited at the exit interview post randomisation and form the cohort of young people followed up at three and 12 months, see Fig. 1.

* Issues raised or discussed only during last consultation, issues discussed at previous consultations were coded as “No”

§ Estimated OR adjusted for socio-economic status of the practice location, practice billing type, sex and age of young people, recruitment method of young people

† Intra-cluster correlation (ICC) estimated using one way analysis of variance; ICC values not shown were truncated at zero

‡ Estimated OR calculated using marginal logistic regression using generalised estimating equations with robust standard errors to adjust for clustering at the clinic level

** Discussed at least one of the two sexual health risks (Contraception and/or protection from STIs)

^††^ For young people aged 17 years old or greater (N=318 in intervention group and N=413 in comparison group)
